# Macroplastic distribution patterns and accumulation in an urbanised Austral subtropical river system

**DOI:** 10.1038/s41598-025-94282-w

**Published:** 2025-03-18

**Authors:** Tatenda Dalu, Collins Oduro, Retang M. Matsimela, Linton F. Munyai, Naicheng Wu, Sydney Moyo, Ross N. Cuthbert

**Affiliations:** 1 Aquatic Systems Research Group, School of Biology and Environmental Sciences, University of Mpumalanga, Nelspruit, 1200 South Africa; 2 Department of Geography and Spatial Information Techniques, Ningbo University, Ningbo, 315211 China; 3 Department of Biological Sciences, Louisiana State University, Baton Rouge, LA 70803 USA; 4 Institute for Global Food Security, School of Biological Sciences, Queen’s University Belfast, Belfast, BT9 5DL UK

**Keywords:** Plastic pollution, Crocodile river, Riparian zone, Transect, Polymer type, Seasonality, Freshwater ecology, Environmental chemistry, Environmental impact

## Abstract

Plastic products have resulted in enormous pollution in many ecosystem types and regions worldwide. The problem is particularly prominent within aquatic environments, where multiple anthropogenic sources (i.e., agriculture, urbanisation, industries, illegal dumping) are common, exacerbated by interconnectedness between aquatic and terrestrial environments and management challenges. Regional disparities are also common within macroplastic research, with a scarcity of knowledge in African freshwaters. Here, by considering seven riparian sites across four seasons, we determined the abundance and distribution of macroplastic litter along the South African Crocodile River system and its associated tributaries. Macroplastics were sorted and classified into various polymer groups, functional origins, and physical forms for each site and season. We hypothesised that macroplastic abundances would be substantial, with differences among sites and seasons, related to differences in human activities along the river shores, particularly during the summer months when recreational activities was high. We observed significant differences in macroplastic abundances and variation across sites and seasons, with a high macroplastic abundance during autumn (338), while lower total numbers of macroplastics unexpectedly collected during spring and summer (243–263). High proportional abundances of plastic bags and film across all sites and seasons were observed, as well as high abundances of polypropylene polymers. Our study serves as a baseline for understanding seasonal abundance and distribution variations in plastic litter and their driving factors in subtropical river systems, which may help to inform management policies. The study further contributes to resolving knowledge gaps in underrepresented regions, providing novel insights into plastic pollution sources, accumulation, and impacts linked with unique socio-environmental contexts. Thus, it bridges critical data gaps, informing targeted interventions and global comparative analyses in plastic waste management.

## Introduction

Since the twentieth century, plastic has been used in rising quantities alongside the growth in human populations, and its single uses have been prominent^1^. The widespread use of plastic has increased due to its numerous advantages^2^. Plastic is affordable, long-lasting, lightweight, abundant, and can be moulded into different shapes^1^. The ongoing accumulation of plastic waste in natural environments has led to its identification as a significant environmental hazard^3^. The growth and impact of plastic pollution can vary across habitat types. Most plastic pollution originates on land^4^, with freshwater acting as conduits for plastic pollution between terrestrial and marine realms.

Macroplastics are large plastic litter items, typically defined as plastic fragments, objects, or waste larger than 5 mm in size^5^. From land, macroplastics can enter rivers through natural factors such as wind^5–8^. Macroplastics can also enter freshwater ecosystems directly through littering, untreated wastewater, sewage systems, and overflowing drains^4,9^. The sources of macroplastic litter accumulation in freshwater are further enhanced by activities such as recreational boating, tourism, and public events, and a significant portion of this problem is credited to the disposal of reusable food containers, food wrappers, and litter from those activities^9,10^.

Once plastic enters waterways, various hydrological factors such as water level, flow velocity, discharge, and polymer characteristics affect their movement, breakdown, and permanence^11–13^. When plastic reaches the river system, horizontal and vertical transportation of litter increases^2^. The horizontal transport of plastic litter occurs due to the river flow velocity, which affects the distance and speed of movement^13,14^. The density, surface area, and particle size of plastics in river systems further enhance vertical transportation^15–17^. The entrapment of plastics in rivers by sediments and plants often leads to increased degradation causing the formation of microplastics, transport of contaminants, habitat disruption, harming of aquatic life, and potential downstream transport of plastics to larger water bodies, ultimately contributing to environmental pollution^5,15,18,19^. On the other hand, low–density results in plastic floating on the river surface, influencing settlement on riverbanks^8,17,20,21^ and re-entrance of plastics into the river system during flood events^22^.

The production of plastic involves chemicals that persist in degradation, resulting in toxic contamination of aquatic ecosystems^23^. The prolonged accumulation of plastic in rivers due to abandonment causes bioaccumulation and biomagnification^17,23^. The detrimental effects related to the accumulation of plastic in aquatic environments cover multiple aspects; these include the ingestion of plastics by organisms, entanglement in polymers, the spillage of toxic additives and consequent buildup of toxins, and the breakdown of plastics into micro or nanoplastics^2,12,24^. The distribution of plastic litter in aquatic ecosystems can also permit transport of non–native species and impacts on aquatic biodiversity^25^.

Land use and land cover changes, particularly urbanization, agricultural activities, and deforestation, have significantly contributed to the introduction of macroplastic waste into aquatic environments such as rivers^8^. Urban areas, with increased plastic waste generation and inadequate waste management systems, serve as primary sources of macroplastic pollution^26^. In agricultural regions, plastic materials like mulch films and goods packaging can be transported by runoff during rainfall events and find their way into river systems^8^. According to Barahona–Segovia et al.^27^, deforestation and land clearance further exacerbate this process by increasing soil erosion and reducing natural barriers, facilitating the transport of macroplastics into river systems. The use of satellite imagery (i.e., Landsat sensors) is crucial for assessing land use and land cover, as it provides large–scale, high–resolution data that can help identify patterns of human activity contributing to the transport of macroplastics into rivers^28,29^. By mapping urban, agricultural, and deforested areas, satellite images enable the detection of potential plastic pollution sources and the pathways through which plastics enter aquatic systems.

Research on riverine macroplastics in Austral subtropical river systems addresses critical knowledge gaps regarding the sources, accumulation, and environmental impacts of plastic pollution in this region. This region largely falls under the developing economy status, with significant urbanisation and limited waste management infrastructure, being a key contributor to global plastic leakage into aquatic ecosystems^30,31^. Previous studies have indicated that Africa is a significant contributor to plastic waste and its mismanagement, with approximately 21% of this litter globally originating from Africa^32^. Despite the complexity of plastic accumulation and the diversity of potential effects, there is an imbalance in the distribution of research efforts across world regions and a lack of recognition of the context–dependency of plastic pollution severity^33^. Most research on riverine plastic pollution has concentrated on rivers in Europe and North America^34^. Despite the subtropical vulnerability, data on the prevalence, composition, and ecological risks of river macroplastics is sparse. To this end, there is a need to close the knowledge gap, as very few studies are conducted on the African continent^35^.

Understanding the seasonal trends of macroplastics in these data–poor regions could direct the intensity of management efforts through space and time. Therefore, the present study investigated the abundance, distribution, and types of macroplastics along an urbanised Austral subtropical river (Crocodile River, Nelspruit City) and its riparian zone in relation to land use activities. In addition, the study further quantified the seasonal and site–specific variation in macroplastic abundance and distribution in relation to land cover activities. We hypothesized that (i) macroplastic abundance would be high during the summer due to increased tourism and activities along the riparian zones compared to other seasons, and (ii) more built-up areas will have high macroplastic densities compared to other land cover classes. This work is novel in that it provides region-specific insights into macroplastic dynamics, enabling targeted mitigation strategies. It aligns with global priorities for plastic pollution management through addressing plastic pollution sources, transport, and accumulation along tributaries, and it aligns with initiatives such as the global plastic treaty, and supports Sustainable Development Goals (SDGs) 6, 11, and 14^36^.

## Material and methods

### Study area

**Fig. 1:**
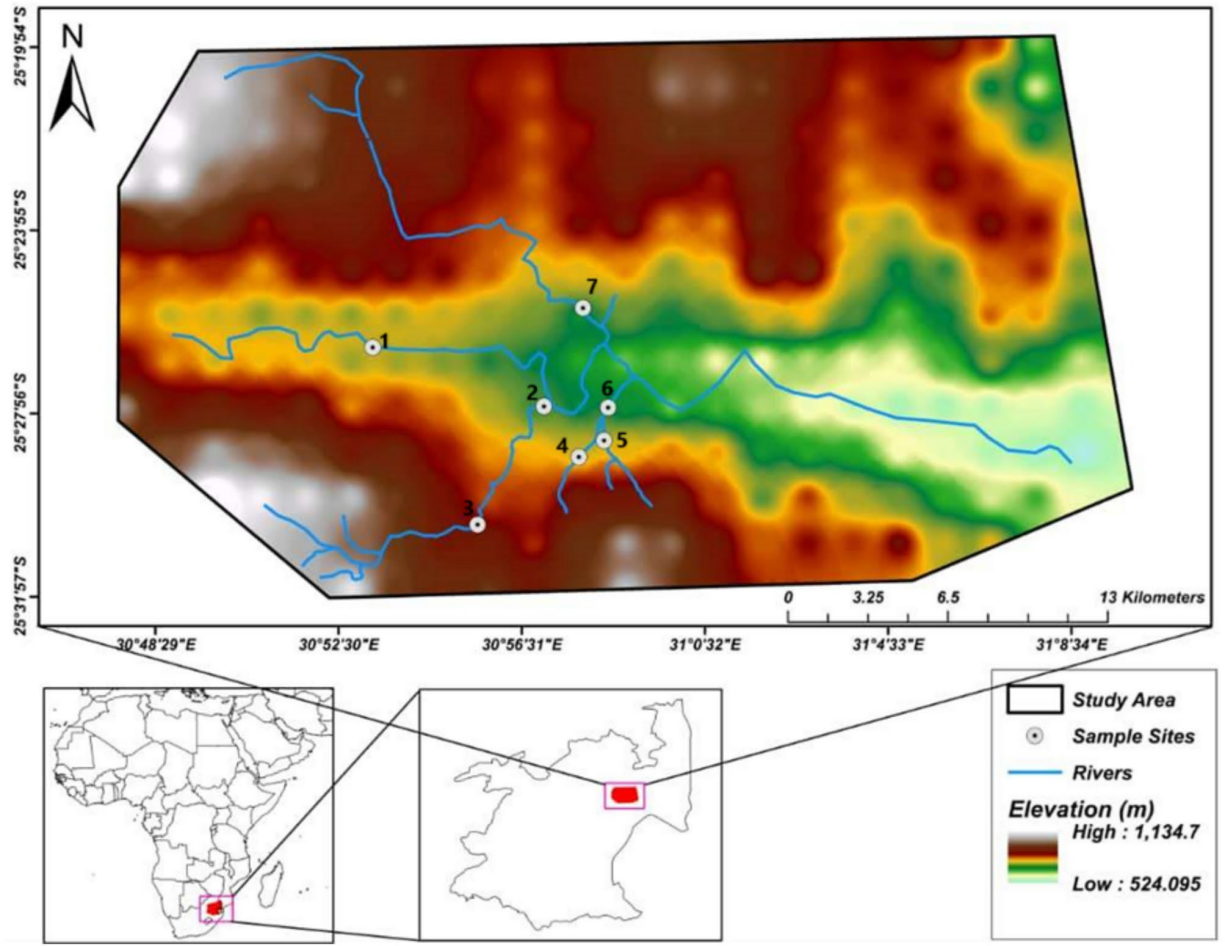
Study sites along the Crocodile River system, Nelspruit City, South Africa. Image created using ENVI software (version 5.3; https://www.nv5geospatialsoftware.com/Products/ENVI) and ArcGIS Pro (version 3.3.2; http://univen.maps.arcgis.com/home/user.html?user=farai.dondofema_univen) with freely available satellite imagery from https://earthexplorer.usgs.gov/. *Source* RSA SG and projection WGS84.

The study was conducted along the subtropical Crocodile River system and its associated tributaries within the City of Nelspruit, Mpumalanga province of South Africa (Fig. 1). The Crocodile River is one of the largest rivers in the province and country and is one of the main tributaries of the Inkomati River system, covering a catchment area of 10 450 km^2^, with a total mainstem river length of 320 km^37^. The mean annual rainfall is 767 mm within the Nelspruit region, with the area receiving an approximate mean rainfall of 9 mm and 131 mm in June and December, respectively^38^. The city’s mean minimum and maximum temperatures are 6 °C (i.e., June, July) and 29 °C (i.e., January, February), respectively^38^.

The Crocodile River catchment was the first water management area in the country to be declared under South Africa’s new National Water Act No. 36 of 1998 as part of the Inkomati Catchment Water Management Area^39^. The river plays an important role as it is utilised for various activities along its length, ranging from water abstraction for domestic and industrial use to subsistence and commercial agriculture and forestry^40^. However, the Crocodile River faces several pollution problems and concerns and is rated amongst South Africa’s top polluted rivers^41,42^.

The study was conducted along the Crocodile River within the Nelspruit City precinct from 7 sites across 4 seasons (Fig. S1). The sites were randomly selected considering issues of accessibility and land cover of the area to represent the anthropogenic stressors within the city based on observations and previous visits to them. Site 1 was impacted by traditional rituals (i.e., sacred spaces used for ceremonies, ancestral worship, and/or spiritual gatherings) and agriculture (i.e., water abstraction, pesticides), sites 2 and 4–7 by sewage spillages, construction, and illegal domestic waste dumping, and site 3 by illegal construction/domestic dumping and traditional ritual activities.

### Research design and sampling

A quantitative method (i.e., riparian surveys) was used to determine the abundance and distribution of macroplastics along the Crocodile River riverbanks from seven randomly selected sites (see Fig. S1; Table S1). This approach allowed for repeated data collection over time, making spatial trend assessments more reliable^43^. Plastic waste was collected from the riparian zone shoreline within a 5 × 5 m quadrat area from both sides of the river system (*n* = 2 per site)^44^ once per each season (Fig. S2). The sampling campaign was conducted in May 2023 (Autumn), July 2023 (Winter), September 2023 (Spring) and November 2023 (Summer). A flow chat of the research strategy is highlighted in Fig. S2.

### Processing of macroplastic samples

The collected macroplastic waste was sorted, washed, and classified into several polymer groups before counting, air drying for 7 days, and weighing in the laboratory. According to Lippiatt et al.^44^, polymer groups are determined by considering their functional origin (e.g., food wrappers, plastic bags) and physical form (i.e., hard, film, foam)^44^. Polymer groups (i.e., LDPE—low-density polyethylene, PET– Polyethylene terephthalate, PS—polystyrene, PVC—polyvinyl chloride, PP—polypropylene, CA—cellulose acetate, HDPE—high–density polyethylene, ABS—acrylonitrile butadiene styrene, PI—polyisoprene, FPM—fluorocarbon–based rubber) were also determined according to Lippiatt et al.^44^ and Plastics Europe^45^ and by further reading the composition list on the items, if present. The collected macroplastic polymers, functional origin, and physical form were counted for each site and season.

### Geospatial analysis

This study further used satellite data for the four seasons (i.e., autumn, winter, spring, and summer 2023) across sites, which were obtained from Landsat 7 ETM + ^46^, to characterise potential pollution accumulation. For multispectral bands on Landsat images, atmospheric corrections were not done since all downloaded images had 5% cloud cover, while radiometric corrections were carried out using ENVI software^47^.

#### Acquisition of images

Several images were downloaded freely from the United States Geological Service (USGS) database^48^, compared and sorted within three days; the best image was selected based on clarity and day of acquisition. Images used were recorded by the sensor in the study area (i.e., autumn, winter, spring and summer; Table 1). This was important as that period had less cloud cover (see Table 1), and all land surface features exhibited consistent reflectance properties irrespective of the year of acquisition. The images acquired covered the study area entirely; hence, mosaicking was unnecessary. The ENVI version 5.3 software^47^ was used to process the images, after which a map was produced in ArcGIS Pro version 3.3.2 on the processed images for spatial data analysis and mapping. Satellite maps and study site visualizations were created using Google Earth Pro (version 7.3).

**Table 1:** Characteristics of the satellite data used in the present study which correspond to the macroplastic sampling campaigns. *Source* USGS^48^.

Capture date	No. of bands	Pixel spacing (m)	Sensor
15 May, 15 July, 15 September, 15 November 2023	7, 4, 2 (3 bands)	30 × 30	ETM + (LE07)

Satellite image attributes.

#### Land use land cover classification

The land use land cover (hereafter referred to as LULC) information was derived from multiband raster images through image interpretation and classification, leveraging current methodologies and technologies^49^. A supervised classification was employed to facilitate the automated grouping of pixels with similar reflectance values into predefined LULC categories^50–52^. Among several techniques for implementing supervised classification, we opted for the widely utilised maximum likelihood classifier, recognised for its efficiency and reliability^53^. This was executed using the ENVI version 5.3 software, reflecting the latest standards in image processing^47^. The maximum likelihood classifier works by assessing the variance and covariance of spectral response patterns, assigning each pixel to the class with which it exhibits a high probability of association. This analysis delineated five primary LULC classes, encompassing agricultural land, built–up areas, bare land, vegetation, and waterbodies adhering to a modernised classification scheme inspired by recent environmental studies^54,55^ (Table 2).

**Table 2:** Land use and land cover classification scheme.

Class	Description
Agricultural land	Land devoted to agriculture and mainly used in the cultivation of rice, maize, plantain, and vegetables is included in this class
Built–up	This class represents residential areas, commercial establishments, industrial zones, roads, and other paved surfaces
Bare lands	The class includes bare lands, rock–strewn, and other soil surfaces devoid of vegetation throughout the year
Vegetation	Areas dominated by dense trees and all vegetative cover
Waterbodies	Open water bodies such as lakes, rivers, and permanent ponds are included in this category

Field validation, supported by satellite navigation (GPS) technology, was conducted post–classification to verify ambiguous areas and refine the LULC categories, highlighting the significance of ground–truthing in contemporary remote sensing analyses^56^. Following the classification process, multi-temporal raster layers representing seasons, autumn, winter, spring, and summer, were produced. These layers facilitated a comprehensive assessment of LULC changes over the specified periods, illustrating the dynamic nature of the landscape and underscoring the importance of continuous monitoring.

#### Accuracy assessment using Google Earth verification

Accuracy assessment (AA) was conducted for the four seasons (i.e., autumn, winter, spring, and summer 2023) using sample points from each classified image using ENVI and ArcGIS, and then by overlaying these sample points on Google Earth Pro for verification. Five hundred ground control points were generated from each class (i.e., agricultural land, waterbodies, vegetation, built–up, and bare lands) for the accuracy assessments, making a total of 2 500 samples. The Google Earth Pro time tool was used to verify by retrospectively identifying the changes that have occurred up to the present. Thereby, we used Google Earth pool of images (satellite imagery) from autumn (May) to summer (November), identifying feature changes and allowing for verification across time. The aim of calculating the accuracy assessment was to determine how accurate the raster maps are, which is normally obtained through percentages. The following formula was used for the accuracy assessment:

$$AA=\frac{ASP}{TSP}\times 100$$

where ASP—number of sample points that accurately fall on each required feature, TSP—number of total sample points generated and AA—accuracy assessment. The higher the AA value, the more accurate the raster map is and the more precise the features are depicted on the map. In this case, the average accuracy assessment for autumn, winter, spring, and summer 2023 was used, with an average accuracy assessment of 95%.

#### Change detection analysis

A change detection analysis was used to ascertain the extent of change in the study from autumn (May) to summer (November) 2023. The change detection statistics for autumn, winter, spring, and summer were obtained from the various months, that is, in terms of area (km^2^) and area (%) for the analysis. This helped in obtaining statistical data on how the land cover has changed over time in terms of features (i.e., agricultural land, waterbodies, vegetation, built–up, and bare lands). Furthermore, the extent of each of the various land use and vegetation types was calculated according to the number of cells that defined them, with cells having less than 50% of a specific vegetation type or land use pattern being excluded^53^.

### Data analysis

A correlation analysis was used to analyse the relationships between land cover patterns and macroplastic abundances using square root transformed data in SPSS version 25. A PERMutational ANOVA (PERMANOVA) was used to calculate differences in macroplastic litter types “community” across sites (i.e., 1–7) and seasons (i.e., autumn, winter, spring, summer), with pairwise comparisons being done for significant (*p* < 0.05) factors. The total number of macroplastic litter ‘species’ in each sample (a measure of γ–diversity) and number of litter ‘species’ in each plot (a measure of α–diversity; i.e., the number of categories of river litter—within–habitat diversity)^57^ was calculated for each site and season^58^. A measure of macroplastic litter ‘species’ turnover inside each site (i.e., the Whittaker β–diversity corresponding to the internal heterogeneity in a ‘community’ or in a site) was calculated as βW = γ/mean α^59^. The Shannon–Wiener diversity index (*H*′) and evenness for macroplastics were calculated according to Battisti et al.^60^. Furthermore, the Shannon–Wiener diversity index (H′) a non–parametric diversity is a measure of diversity in a community and/or ecosystem. It quantifies the uncertainty in predicting the “species” identity of an individual randomly selected from the community and takes into account both the number of “species” (richness) and their relative abundance (evenness) and was calculated as follows:

$${H}{\prime}=-\sum fr\times \text{ln}(fr)$$

where *fr* is the relative frequency of each macroplastic particle/colour ‘species’. Evenness index (*E*) was calculated as:

$$E = {\text{H}^\prime \mathord{\left/ {\vphantom {H {H_{{\max }} }}} \right. \kern-\nulldelimiterspace} {\text{H}^\prime_{{\max }} }}$$

where *H*′_max_ = lnS.

A two–way ANOVA was used to assess land use classes, macroplastic diversity matrices, abundances, polymers, and Whittaker β–diversity within sites (i.e., 1–7) and seasons (i.e., winter, spring, autumn, summer). Tukey’s post–hoc analysis was conducted for significant (*p* < 0.05) variables to see which sites drove the differences.

## Results

### Land use and land cover patterns

**Fig. 2:**
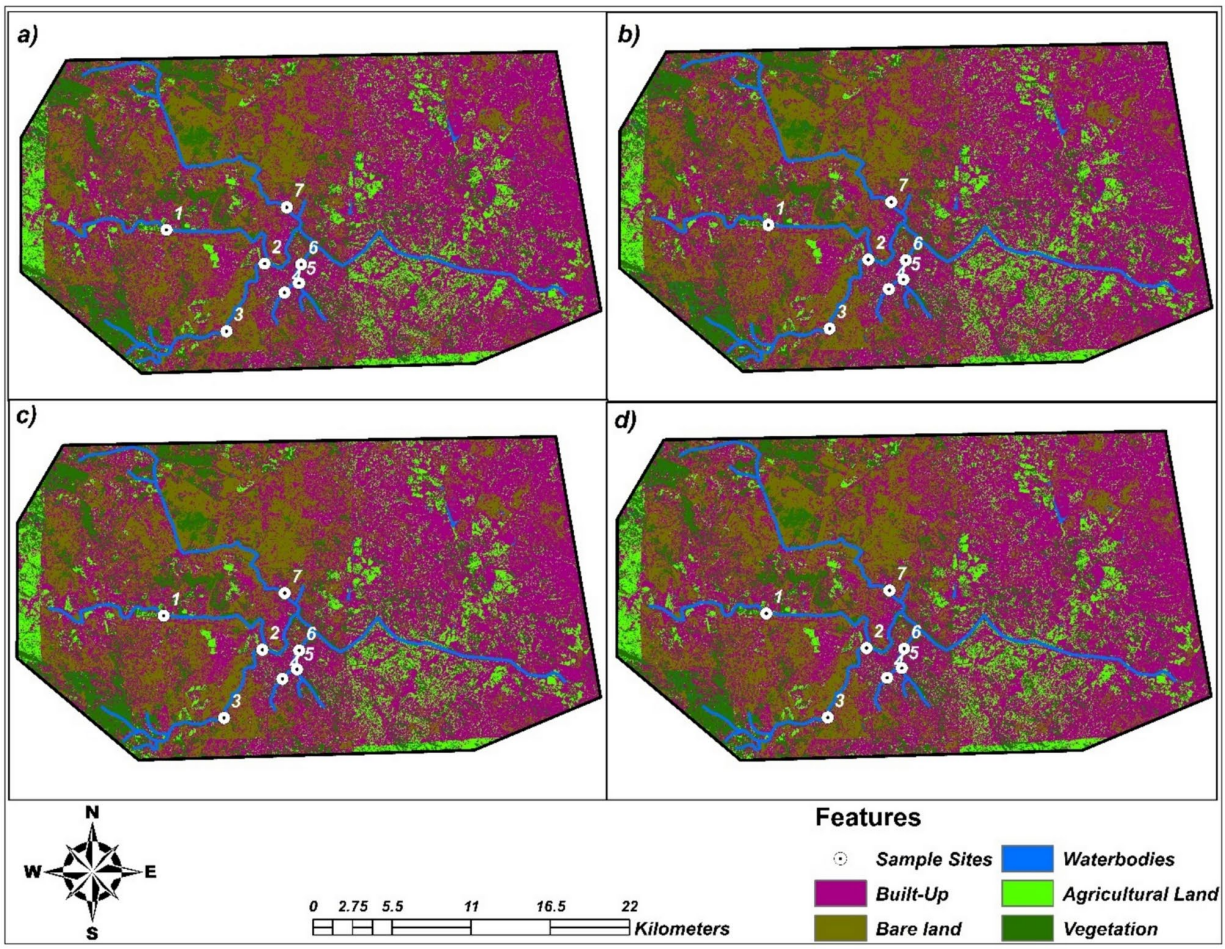
Land use and land cover patterns in: (**a**) autumn, (**b**) winter, (**c**) spring, and (**d**) summer within the Nelspruit city region, Mpumalanga province of South Africa. Image created using ENVI software (version 5.3; https://www.nv5geospatialsoftware.com/Products/ENVI) and ArcGIS Pro (version 3.3.2; http://univen.maps.arcgis.com/home/user.html?user=farai.dondofema_univen) with freely available satellite imagery from https://earthexplorer.usgs.gov/. *Source* RSA SG and projection WGS84.

Built–up areas were the dominant land use (> 50% cover), with waterbodies being the least common land cover (< 0.5%; Table 3; Fig. 2). No significant differences (*p* > 0.05) were observed for the various land use and cover types across seasons. No significant differences in relationships (Correlation, *p* > 0.05) were observed for the different between land cover types and macroplastic abundances. Built–up areas (*r* = 0.80, *p* = 0.201), waterbodies (*r* = 0.24, *p* = 0.833), and vegetation (*r* = –0.76, *p* = 0.245) showed a positive and non-significant relationship with macroplastics, whereas bare lands (*r* = –0.64, *p* = 0.422) and agriculture lands (*r* = − 0.60, *p* = 0.389) showed negative and non-significant relationships with macroplastics.

**Table 3:** The mean percentage (%) land use and cover types for the Nelspruit region, South Africa found across the different sites.

Land cover type	Autumn	Winter	Spring	Summer
Bare lands	23.3	23.4	23.1	22.7
Built up	50.3	50.5	50.7	50.9
Agriculture lands	11.4	11.2	11.4	11.5
Waterbodies	0.38	0.55	0.39	0.46
Vegetation	14.6	14.4	14.4	14.5

### Macroplastic distribution

**Fig. 3:**
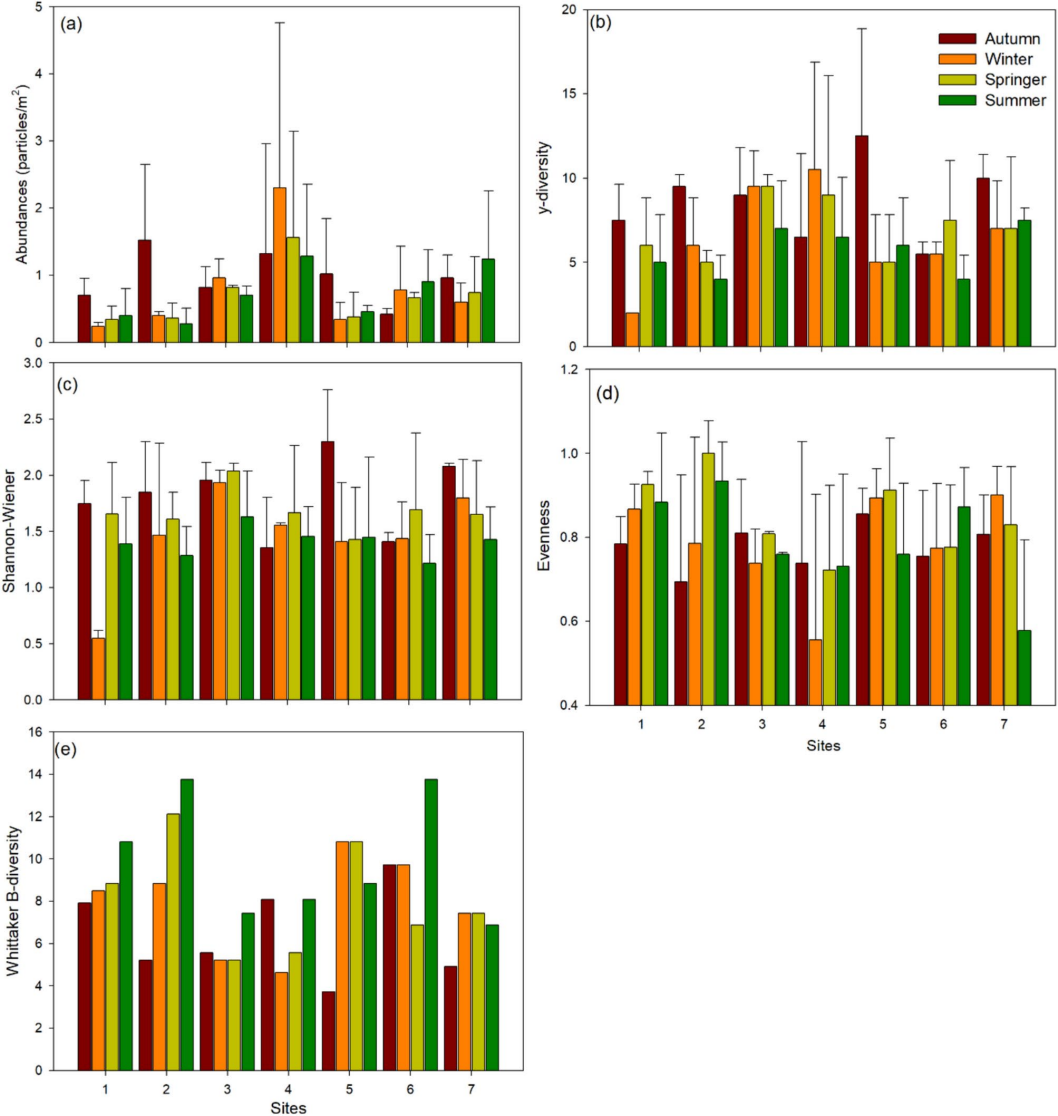
Mean macroplastic abundance and functional groups (**a**) macroplastic abundance (**b**) ‘litter species’ (γ–diversity), (**c**) Shannon– Weiner index (**d**) Evenness (**e**) Whittaker β– diversity for four seasons along the Crocodile River system.

A total of 1 134 macroplastic litter were collected during the entire study period, with 338, 290, 243, and 263 particles being collected during autumn, winter, spring, and summer, respectively (Fig. 3a). High macroplastic quantities were recorded at site 4 (range 1.28–2.3 particles m^−2^) throughout the study, with site 1 (range 0.24–0.40 particles m^−2^), 2 (range 0.28–0.40 particles m^−2^) and 5 (range 0.34–0.44 particles m^−2^) having relatively low macroplastic densities during winter, spring and summer (Fig. 3a). The γ–diversity showed a variable trend across study sites and seasons, with high and low diversity being observed in site 5 during autumn (mean = 12.5) and site 1 during winter (mean = 2), respectively (Fig. 3b). The Shannon–Wiener diversity index had no clear trends across seasons, with winter site 1 having low diversity index values (mean 0.55 ± 0.07) (Fig. 3c). Evenness was high for all seasons and sites, with the exception of winter site 4 (mean 0.56 ± 0.35) and summer site 7 (mean 0.58 ± 0.21) (Fig. 3d). The Whittaker β–diversity for all seasons ranged from 4.62 to 13.75 (Fig. 3e). No significant differences (*p* > 0.05) were observed for all diversity indices, except for the Shannon–Wiener index which was significantly different among seasons (F = 3.151, df = 3, *p* = 0.040) (Table 4).

**Table 4:** Two–way ANOVA of the macroplastic litter diversity indices and polymers from Crocodile River.

Variable	Sites			Seasons		
	DF	F	*p*	DF	F	*p*
Diversity metrices						
Litter ‘species’ γ–diversity	6	1.797	0.136	3	2.210	0.109
Abundances	6	1.878	0.120	3	0.598	0.622
Shannon–Wiener diversity index	6	1.923	0.112	**3**	**3.151**	**0.040**
Evenness	6	1.232	0.320	3	0.516	0.675
Whittaker β–diversity	6	0.046	1.000	3	0.043	0.988
Polymer abundances						
Acrylonitrile Butadiene Styrene (ABS)	6	0.396	0.875	3	1.857	0.160
Cellulose acetate (CA)	6	0.504	0.800	3	0.689	0.567
Flourocarbon–based rubber (FC)	6	1.412	0.245	3	1.677	0.194
High–density polyethylene (HDPE)	6	1.167	0.352	3	0.257	0.856
Low–density polyethylene (LDPE)	6	0.629	0.705	3	0.652	0.588
Polyisoprene	6	0.918	0.497	3	2.201	0.110
Polyethylene terephthalate (PET)	**6**	**2.494**	**0.046**	3	0.445	0.722
Polypropylene (PP)	6	0.435	0.849	3	1.338	0.282
Polystyrene (PS)	6	2.170	0.076	3	1.145	0.348
Polyvinyl chloride (PVC)	6	0.652	0.688	3	0.163	0.920
Polymer weight						
Acrylonitrile Butadiene Styrene (ABS)	6	0.957	0.471	3	2.921	0.051
Cellulose acetate (CA)	6	0.850	0.543	3	1.158	0.343
Flourocarbon–based rubber (FC)	6	0.899	0.509	3	0.183	0.907
High–density polyethylene (HDPE)	6	0.774	0.597	3	1.744	0.181
Low–density polyethylene (LDPE)	6	0.767	0.602	3	1.723	0.185
Polyisoprene	6	0.948	0.477	3	0.649	0.590
Polyethylene terephthalate (PET)	6	1.132	0.370	3	1.528	0.229
Polypropylene (PP)	6	1.692	0.160	3	1.984	0.139
Polystyrene (PS)	6	1.371	0.261	**3**	**3.187**	**0.039**
Polyvinyl chloride (PVC)	6	0.711	0.643	3	1.732	0.183

Bold values indicate significant differences at *p* < 0.05, DF—degrees of freedom.

### Macroplastic litter functional type and polymer groups

Based on PERMANOVA analysis, no significant differences were observed across sites (Pseudo–F = 1.255, df = 6, *p*(Monte–Carlo) = 0.127) and seasons (Pseudo–F = 1.432, df = 3, *p*(Monte–Carlo) = 0.088) for macroplastic functional types. The litter collected across all four seasons across the different study sites varied according to functional types, polymers, and abundances (see Tables S2–S5 and 5). Overall, the winter season had more diverse macroplastic types compared to all other seasons. The most dominant and abundant macroplastic litter collected across all seasons was plastic bags, plastic bottles and food wrappers [combined mean range: autumn 34.4–68.2%; winter 11.5–90.0%; spring 29.2–51.1%; summer 38.2–71.6%] (Table 5). Food wrappers were the dominant plastic type in spring and autumn (Table 5). The least observed macroplastic litter collected across all seasons were bubble blowers, peanut butter containers, red tape, cigarette filters and packages, and yoghurt containers [combined mean range: autumn 0.0–11.1%; winter 0.0–13.8%; spring 0–2.1%; summer 0–5.0%] (Table S2–S5). Site 1 during winter had the least diverse macroplastic litter analysed, with plastic bags being the most collected macroplastic.

**Table 5:** Mean (± standard deviation) macroplastic litter type, polymer, and abundance (% per 25 m^2^) found along the Crocodile River across seasons (*n* = 14 per season).

Plastic fragments	Type	Polymer group	Autumn	Winter	Spring	Summer
Appliance part 1	Hard	ABS	3.5 ± 4.8	3.8 ± 8.3	1.9 ± 5.9	1 ± 4.1
Appliance part 2	Hard	LDPE	0.5 ± 1.5	0.6 ± 1.6	0.2 ± 0.9	0.5 ± 2
Bottle cap 1	Hard	HDPE	**13.2 ± 10.7**	**7.8 ± 10.9**	**12.4 ± 12.6**	**5.2 ± 8.9**
Bottle cap 2	Hard	HDPE		0.4 ± 1.8		2.1 ± 8
Bubble blower	Hard	PP		0.2 ± 0.9		
Cider plastic container	Film	PS			1.7 ± 3.8	0.3 ± 0.9
Cigarette filter	Hard	CA	1.5 ± 5.9	2.1 ± 5.9	3.0 ± 10.0	
Cigarette pack	Film	PP	0.1 ± 0.4	1.4 ± 3.2	2.1 ± 5.4	0.7 ± 2.6
Deodorant	Hard	HDPE	0.9 ± 2.9	2.2 ± 6.2		0.3 ± 1.3
Detergent bottle 1	Film	HDPE	1.1 ± 3.1			
Detergent bottle 2	Hard	HDPE	0.6 ± 2.0		0.4 ± 1.7	
Diapers	Foam	Mixed	0.2 ± 1.0			
Elastic Band	Hard	LDPE			1.4 ± 5.3	
Floss	Hard	HDPE			1.4 ± 5.3	
Food container 1	Foam	PS	2.4 ± 3.8	**5.7 ± 13.4**	0.8 ± 2.1	4.6 ± 9.2
Food container 2	Foam	PS	**5.2 ± 6.7**	**12.5 ± 20.1**	**5.8 ± 7.4**	3.1 ± 6.3
Food wrapper	Film	PS	**10.2 ± 14.1**	**19.3 ± 22.8**	**16.4 ± 16.2**	**27.2 ± 20.1**
Furniture wrapper 1	Film	LDPE	1.0 ± 2.7	2.4 ± 6.9	**5.3 ± 12.2**	**6.7 ± 10.6**
Furniture wrapper 2	Foam	PS	0.8 ± 2.4	2.7 ± 7.2		
Light bulb holder	Hard	PVC			0.3 ± 1.2	
Gloves	Hard	PVC	1.7 ± 3.3			0.5 ± 2
Glue container	Hard	PET			0.1 ± 0.3	2.3 ± 8.9
Juice box	Hard	PP		0.0 ± 0.2		
Lunchbox lid	Hard	PP			0.4 ± 1.6	
Mask	Foam	PP			0.3 ± 1.2	
Mat	Hard	LDPE	0.2 ± 1.0			
Medicine bottle	Hard	HDPE	0.5 ± 1.8		1.0 ± 4.1	
Milk container	Hard	PP	1.4 ± 2.6			2.4 ± 6.8
Motor oil bottle	Hard	HDPE		0.8 ± 2.0		0.5 ± 2
Jug	Hard	HDPE	0.2 ± 0.7			
Packaging plastic	Film	LDPE			0.4 ± 1.7	1.4 ± 2.8
Peanut butter container	Hard	PET		0 ± 0.2		
Perfume bottle	Hard	HDPE	0.2 ± 0.8	0.5 ± 2		3.6 ± 9.7
Pipe	Hard	PVC	2.2 ± 6.7			
Plastic bags	Film	LDPE	**20.1 ± 17.1**	**16.3 ± 22.6**	**8.7 ± 8.6**	**21.9 ± 19.4**
Plastic bottles	Hard	PET	**19 ± 11.8**	**11 ± 13.8**	**19.5 ± 14.5**	**12.5 ± 13.3**
Plastic cup 1	Film	LDPE	1.2 ± 3.1	0.4 ± 1.8		
Plastic cup 2	Foam	PS	1.4 ± 5.3	1.0 ± 3.0	0.3 ± 1.3	0.1 ± 0.5
Plastic cutlery	Hard	PP	1.5 ± 4.5		1.5 ± 3.5	
Plastic hanger	Hard	ABS			1.4 ± 5.3	
Plastic lid	Hard	PP	0.6 ± 1.2	0.4 ± 1.8		
Plastic rope	Film	PVC	0.8 ± 1.6	0.3 ± 1.4	1.0 ± 2.7	
Plastic straw	Film	PP		0.2 ± 0.7	0.3 ± 1.1	
Pregnancy test stick	Hard	HDPE			0.3 ± 1.1	0.1 ± 0.5
Red tape	Hard	PVC		0.0 ± 0.2		
Rubber band	Hard	IR		0.7 ± 2.6		
Sack	Film	PP	2.5 ± 5.2		3.2 ± 8.9	1.2 ± 3.2
Sanitizer bottles	Hard	PP			0.8 ± 3.3	
Shaver	Hard	HDPE	0.1 ± 0.6			
Shoe	Hard	LDPE	1.2 ± 2.5		1.0 ± 2.6	
Shoe sole	Hard	PVC			1.4 ± 5.3	0.5 ± 2.0
Soap wrapper	Film	LDPE	0.8 ± 2.4	2.7 ± 6		
Strapping tape	Hard	PP	1.0 ± 2.4	1.2 ± 3.5	0.5 ± 2.2	
Toothpaste tube	Hard	LDPE	0.1 ± 0.6			
Umbrella	Film	PET		0.3 ± 1.4		
Vaseline container	Hard	PP		0.7 ± 2.9	0.2 ± 0.9	
Yogurt container	Hard	PS	0.1 ± 0.4	0.5 ± 1.9	2.6 ± 8.8	0.1 ± 0.5

Abbreviations: ABS—acrylonitrile butadiene styrene, CA—cellulose acetate, LDPE—low density polyethylene, PET—Polyethylene terephthalate, PS—polystyrene, PVC—polyvinyl chloride, PP—polypropylene, HDPE—high–density polyethylene, IR—isoprene rubber, values in bold indicate > 5% mean relative abundances.

**Fig. 4:**
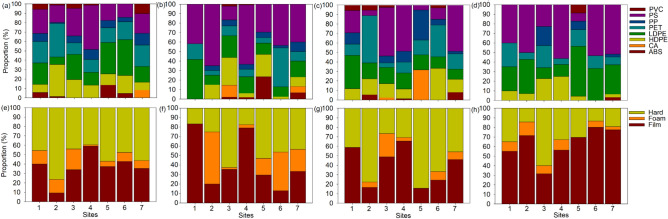
The macroplastic polymer group (**a**–**d**) and physical form (**e**–**h**) over four seasons (i.e., autumn, winter, spring, summer) for seven sites along the Crocodile River system excluding mixed polymer and isoprene which were only observed once. Abbreviations: LDPE—low density polyethylene, PET– Polyethylene terephthalate, PS—polystyrene, PVC—polyvinyl chloride, PP—polypropylene, CA—cellulose acetate, HDPE—high–density polyethylene, ABS—acrylonitrile butadiene styrene, PI—polyisoprene, FPM—fluorocarbon–based rubber.

Macroplastic litter comprised eight polymers that showed variations in abundance among sites and seasons (Fig. 4a–d). Polystyrene (PS) was one of the abundant plastic polymers (range 5.3–53.3%) across sites and seasons. A total of 94 items was recorded for the autumn season and 76 items were collected for the spring season, with the least dominant plastic polymer being polyisoprene (PI) with 4 litter items during winter and 1 plastic item during the spring season. For all the sites, the spring and autumn had high abundances of macroplastics in comparison to the winter season in all plastic polymer types. The PS, PP, PET, and LDPE were most dominant plastic polymer in terms of abundances across all sites for all seasons, while, cellulose acetate (CA), fluorocarbon–based rubber (FPM), and PI were relatively low in terms of abundances across all seasons (Fig. 4a). For all seasons, PS was the most dominant in terms of abundance, followed by PP, with PI being the least dominant. Only PET abundance showed significant differences among sites, and PS weight among seasons (Table 4). The physical form was dominated by film and hard, with foam being the least dominant physical form with no clear seasonal variation patterns (Fig. 4e–h).

## Discussion

Macroplastic pollution is a global phenomenon, but remains geographically and environmentally biased, lacking investigation in southern African freshwaters. This study aimed to investigate the abundance and distribution patterns of macroplastic litter along the Crocodile River, South Africa. In contrast to our a priori hypothesis, our data showed no significant differences in macroplastic litter abundance across sites and seasons, indicating that the problem is widespread spatiotemporally. The observed high levels of PS-based polymers suggest a significant presence of human activities, such as washing, sewage discharge/spillage, and solid waste disposal/dumping along the Crocodile River throughout the year. These activities contribute to an increased likelihood of macroplastic litter accumulation in the riparian zones of the river. Overall, these results suggest that management strategies should operate year-round to remove plastics from riparian habitats. Targeting these areas could thus result in the removal of most plastic litter from river systems. Studies on macroplastic distribution in urbanised subtropical rivers often align with findings linking LULC to pollution^61,62^. Urban areas typically show higher macroplastic accumulation, consistent with global trends, while natural landscapes exhibit lower levels, emphasizing urbanisation’s role in plastic pollution.

The use of Landsat Enhanced Thematic Mapper (ETM +) for determining land use and land cover has proven to be an effective tool in environmental monitoring due to its capacity for capturing large–scale spatial data over time. However, studies such as the current one indicate that the relationship between land use and land cover (LULC) and river macroplastic distribution may not always be significant^63,64^. In such cases, this disconnect could be attributed to various factors, such as the localized nature of plastic pollution, inefficient waste management practices or the influence of hydrological processes, which might overshadow the impacts of LULC. For example, while urban areas typically contribute to more macroplastics, the absence of a clear correlation between LULC patterns and macroplastic presence in the Crocodile River and its tributaries might be due to irregular plastic waste transport mechanisms, such as wind or stormwater drainage, or the presence of non–point sources like illegal dumping by community members from Nelspruit residential areas. Additionally, vegetation and sediments along riverbanks may act as physical barriers, trapping plastics before they can disperse further downstream^18,65,66^. Indeed, riverbank and riparian plastics can act as long-term indicators of river plastics, with high trapping efficiency by trees, shrubs and reeds which can block 85% of the total macroplastic entrapped by plants in South European rivers^5^. Similarly, Gallitelli and Scalici^5^ using a global dataset demonstrated how vegetation play a pivotal role in entrapping plastics across spatiotemporal scales, and they observed high plastic densities on plants rather than in the adjacent water area.

Human activities along the riverbank, including littering by visitors, contribute significantly to plastic pollution. Human activities are, however, not the only contributors of plastic into the river, but natural processes such as rainfall and wind^40^ also contribute to the accumulation and distribution of macroplastics. Ryan and Perold^67^ indicated that litter loads peaked during rain events downstream (i.e., river mouth) and decreased after the first rainfall event, presumably due to flushing of accumulated litter. Garello et al.^68^ showed that the influence of wind intensity on plastic litter transport was comparatively negligible in Paraná River, Argentina, with water flow having a greater capacity to remobilise and transport plastic debris compared to wind. It is challenging to distinguish between litter directly deposited by visitors and plastic litter washed onto the shorelines by wind or water flow^69^. Laverre et al.^70^ highlighted that remediation actions must be taken especially on rainy days and target small litter to significantly limit macroplastic inputs from rivers to the sea, as 73% of plastic are released during rain events on the Têt River (NW Mediterranean Sea).

The dominant macroplastic type observed in this study was plastic bags, which persisted across all seasons. Indeed, similar to this study, research on other river systems has found that plastic bags were the most dominant macroplastic collected^71^. According to that same study, the prevalence of plastic bags is closely linked to economic activities, including market operations, domestic waste management, and recreational activities along the riverbanks, all of which contribute to the continuous presence of plastic bags in the environment. Given that the river is located within an urban area, our analysis suggests that the river predominantly accumulates macroplastics because of direct human activities in the surrounding area, such as recreational fishing, illegal dumping, and run-off from urban and industrial areas. While not accounted for in this study, household distance from riparian zones is another factor that may explain macroplastic concentrations. Given that many households are situated at proximity to the river sites (distance range 20.8–145.1 m), household density near the river is likely to influence macroplastic accumulation directly or indirectly, depending on the income status^72^.

Despite unevenness in their relative contributions, a wide range of macroplastic types and polymer forms were observed in the samples collected from different seasons and sites. Most of these plastics can be associated with household and recreational activities, as Nelspruit is an urban area^40^. Plastic bags, plastic bottles, and food wrappers were the most identified sources for this study, similar to other investigations^73,74^. This dominance of plastic bags, plastic bottles, and food wrappers was consistent across all four seasons. This could be attributed to inadequate solid waste management and lacking general human pro–environmental behaviours, leading to their persistent use and accumulation throughout the year. The fluctuations in the overall quantities of plastic items observed concerning Whittaker β– and γ–diversity across the four seasons can be ascribed to differences in deposition patterns resulting from relevant micro–geographical and environmental influences, including season–dependent recreational activities^60^. Moreover, natural processes such as wind and rainfall increase plastic litter from inadequately managed dump sites and residential areas to bodies of water^36^. These transportation methods then contribute to the accumulation of plastic bags as identified for this study in all seasons, depending on their weather patterns.

The categorisation of plastic polymer types is useful as it enhances the ability to develop predictions regarding the origin of plastic pollutants and assists in establishing whether they arise from the breakdown of macroplastic components that originate from nearby industrial or recreational activities^2^. This study’s plastic polymer type results showed that the most dominant polymers were PP and PS (Fig. 3a), and these characteristics were generally consistent across sites and seasons, with a few exceptions. A considerable fraction of macroplastics comprised food wrappers, bags, and disposable foam food containers. Claessens et al.^75^ and Plastics Europe^76^ show how polypropylene (PP) and PS have been well documented as commonly used materials in food packaging. However, it is important to highlight that PP has been frequently seen in packaging uses, such as bottles, beverage caps, bags, and home appliances or devices^77^.

Physical forms of plastics were also identified for this study, with film being the most dominant. Most of the film macroplastics collected in this study were from the PP polymer. Film plastics are widely used because they are light in weight, slow to degrade, and reusable^1^, while packaging bags are one of the most widely–-used plastic films, as observed in this study, similar to Rohaningsih et al.^78^. Film plastics have also been found to have detrimental effects on aquatic habitats due to their potential ingestion by animals such as fish, birds and macroinvertebrates, leading to physical and chemical consequences^2^. Consequently, the Crocodile River experienced a significant prevalence of film plastic pollution. These macroplastics undergo disintegration, forming small fragments that persistently endanger aquatic animals and confer health hazards to individuals utilising the river’s water resources.

Conducting a macroplastic study along a small section of a major river system with a few sampling sites poses several limitations such as the spatial representativeness, whereby a limited number of sampling sites may fail to capture the variability of macroplastic distribution across the river system, which can be influenced by factors such as hydrology, geomorphology, land use, and proximity to pollution sources^79^. This restricted scope limits the ability to generalise results for the entire river system. Additionally, site accessibility and selection biases, although randomly selected, may influence results, potentially overlooking key hotspots of plastic pollution. Transient events such as floods and festivals can temporarily increase plastic pollution, but in most instances are not aligned with the sampling periods. Furthermore, a small-scale study may not adequately capture downstream impacts, including plastic transport to marine environments. Focusing solely on macroplastics while excluding microplastics may further miss smaller particles, which are equally or more critical for understanding long-term environmental and ecological impacts of plastics. Finally, logistical and resource constraints, inherent in limited-site studies, can hinder efforts to develop comprehensive management strategies. To address these challenges, future studies should incorporate more extensive spatial and temporal sampling to ensure robust and representative data^80^.

## Conclusions

This study assesses plastic pollution characteristics across land uses in a subtropical river system, aiming to inform waste management policies, promote conservation, and raise public awareness. We found that plastic bags, plastic bottles and food wrappers were the most dominant macroplastics. Snapshot surveys such as this can potentially drive sustainable practices, regional environmental strategies, and global collaborations to address plastic pollution and its socio-environmental consequences. Land use and land cover analysis, mainly through satellite-based approaches such as Landsat ETM + , can provide a crucial framework for identifying potential sources and accumulation of riverine macroplastics. The abundance and distribution of macroplastic litter in terms of their functional group, physical shape, and polymer group indicated potential for a substantial amount of dispersion and, thus, consistency between seasons and sites. Nevertheless, this study demonstrated that the dominant macroplastic litter along the Crocodile River could be due to recreational activities, settlements, and illegal dumping. More research is needed to fully understand the origins, fate, impacts, and temporal fluctuations linked to macroplastics in this river and other data-scarce regions. The complexity of plastic pollution dynamics requires a multifaceted approach that incorporates waste management practices, hydrological patterns, and environmental factors to comprehensively assess macroplastic distribution. Thus, it bridges critical data gaps, informing targeted interventions and global comparative analyses in plastic waste management. Findings can guide targeted clean-ups, inform policy on plastic waste management, and enhance public awareness campaigns. Furthermore, we need to improve available waste management infrastructure (as well as finding alternative laundry facilities and improved sewage treatment), so the macroplastic does not end up in rivers and on riverbanks. Thus, implementing stormwater filtration and promoting biodegradable alternatives can reduce macroplastic pollution locally and serve as a model for similar urban river systems globally.

## Supplementary Information

Supplementary Information.

## Data Availability

The datasets generated and/or analysed during the current study are not publicly available as they are part of larger study that is currently on–going but are available from the corresponding author on reasonable request.
