# Supplementary material for: Macroplastic distribution patterns and accumulation in an urbanised Austral subtropical river system
Source: Sci Rep. 2025 Mar 18;15:9231. doi: 10.1038/s41598-025-94282-w (PMC11914210; doi:10.1038/s41598-025-94282-w)
Supplement: Supplementary file 1 — Supplementary Information. [file 41598_2025_94282_MOESM1_ESM.docx]

**Supplementary files**


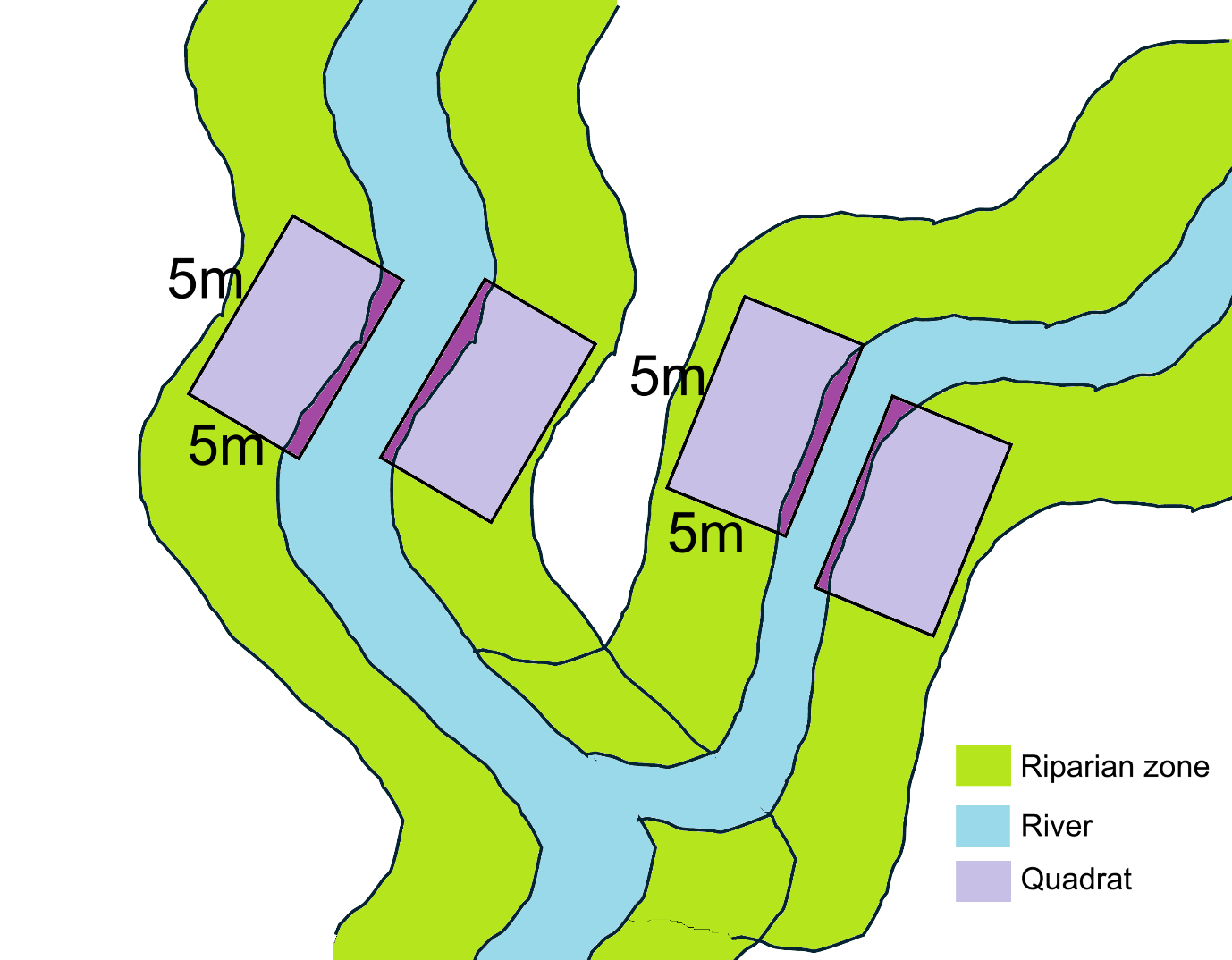


**Figure S1** Macroplastic sampling strategy along the Crocodile River system


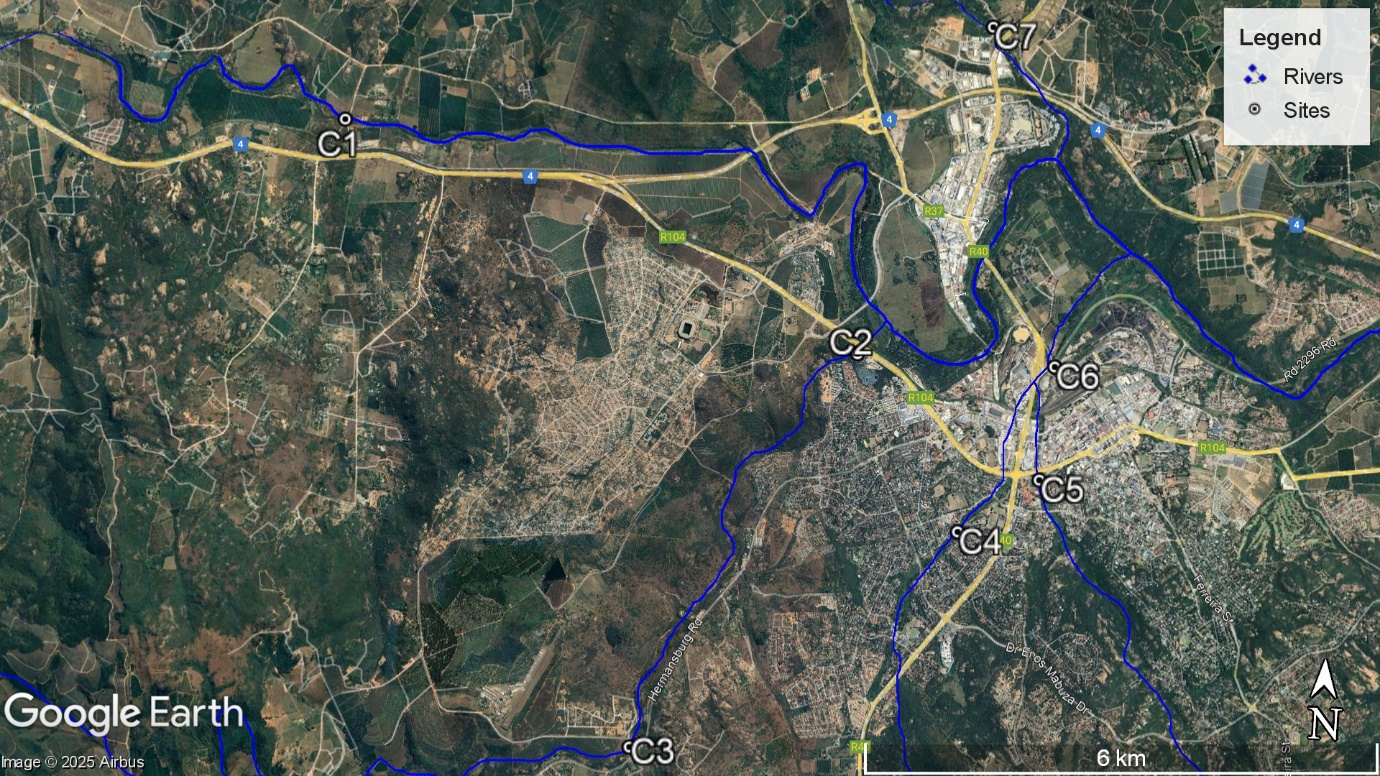


**Figure S2** Enlarged satellite map of sampling sites showing land cover classifications (refer to Table S1 for details). Image created using Google Earth Pro (version 7.3) based on freely available satellite imagery courtesy of Google Earth.


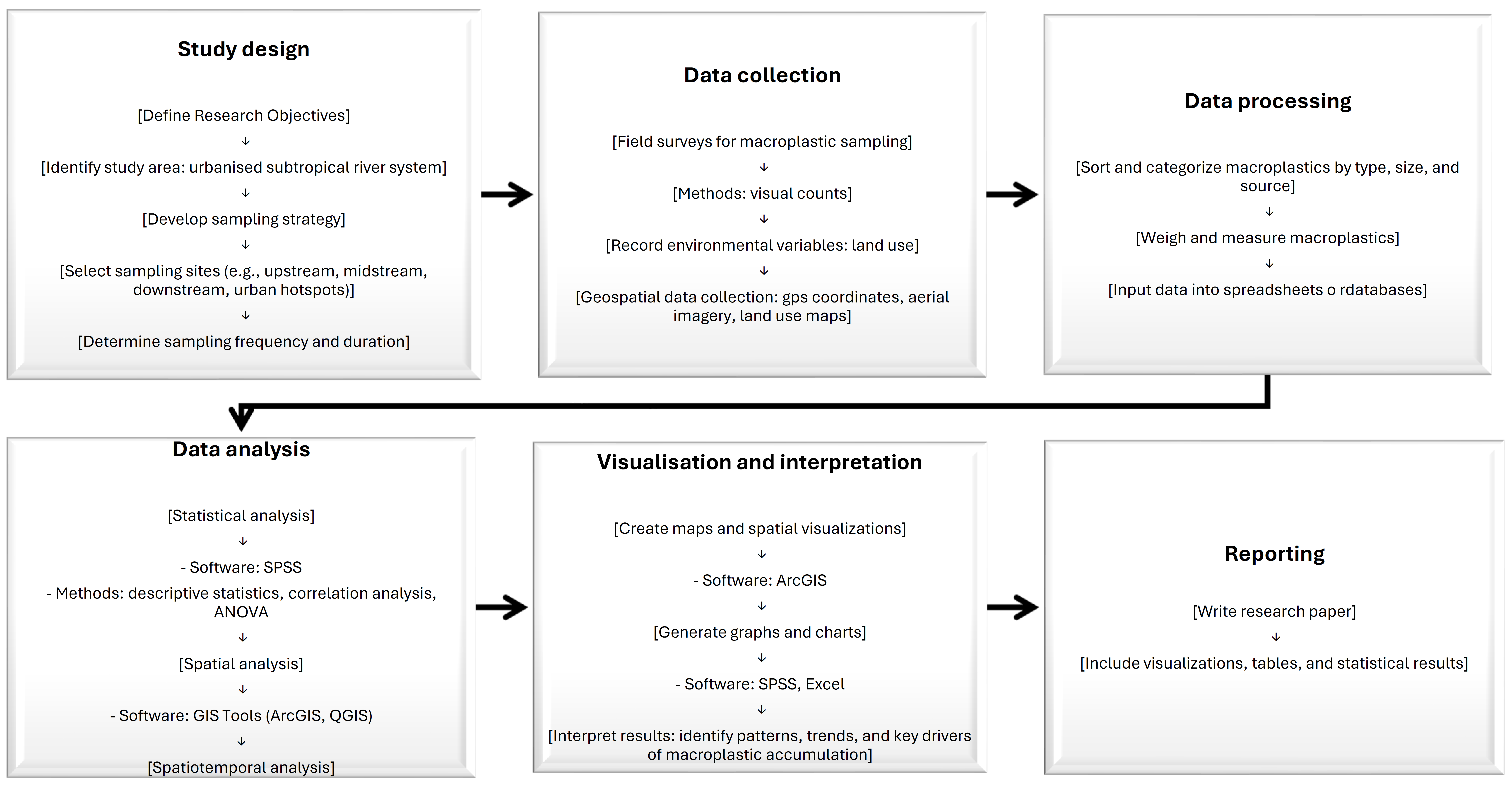


**Figure S3** Flow chat highlighting important steps of the study

**Table S1**. Detailed site descriptions and geographical locations (coordinates) of each site visited for water and sediment sample collection

| **Site name** | **River** | **Site description** | **Coordinates** | **Pictures** |
| --- | --- | --- | --- | --- |
| 1 | Crocodile River (upstream) | Agriculture activities along the system and a ritual activity site. The site consists of dense habitats of reeds and other aquatic vegetation. During period of heavy rainfall, the site could be impacted by runoff water from the nearby farms. | –25.441338°, 30.887864° | 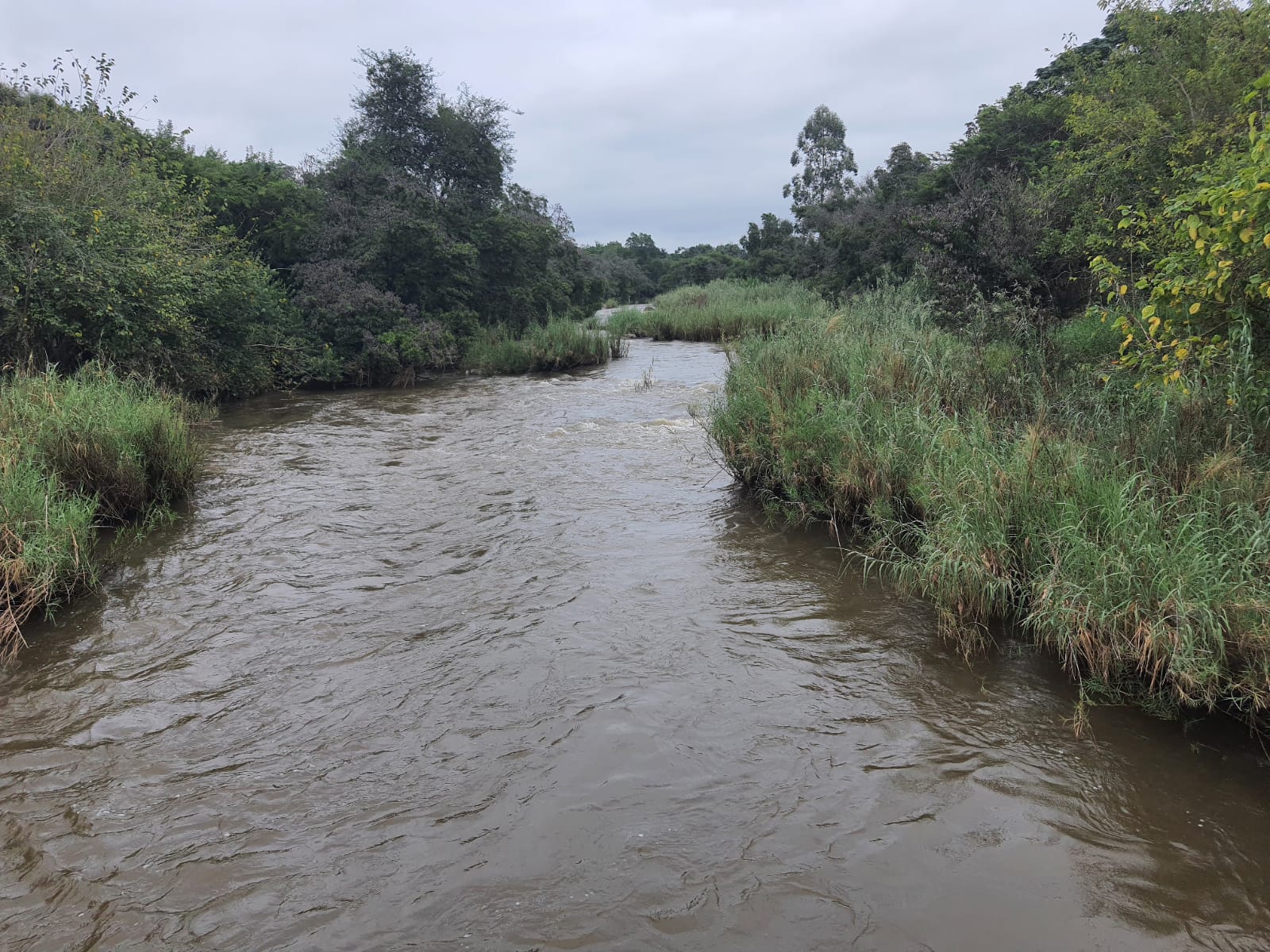 |
| 2 | Gladdespruit (downstream) | This site is located along the new N4. The water flowing into the river is mostly contaminated by the sewage overflow that is coming from the blocked sewage pipes that continuously discharge sewage into the system. at this site, there are some evidence of people making laundry and some even and fishing. | –25.462341°, 30.949689° | 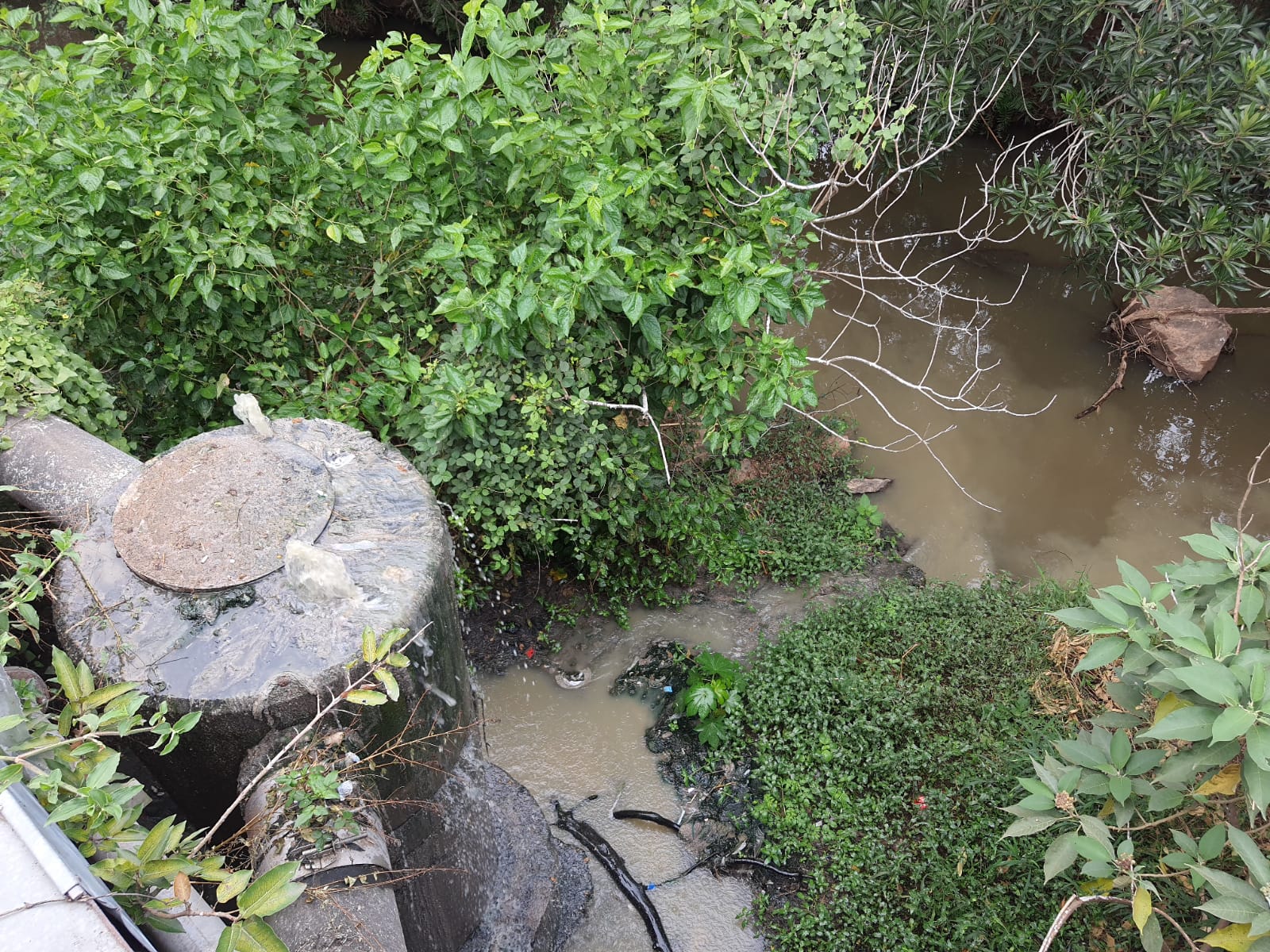 |
| 3 | Gladdespruit (upstream) | This site is highly impacted from several anthropogenic activities of rituals, illegal domestic, and construction rubble dumpsite. It is evident that the activities of this site have ecological and physical impacts on the vegetation and habitat cover of this site. | –25.506185°, 30.925794° | 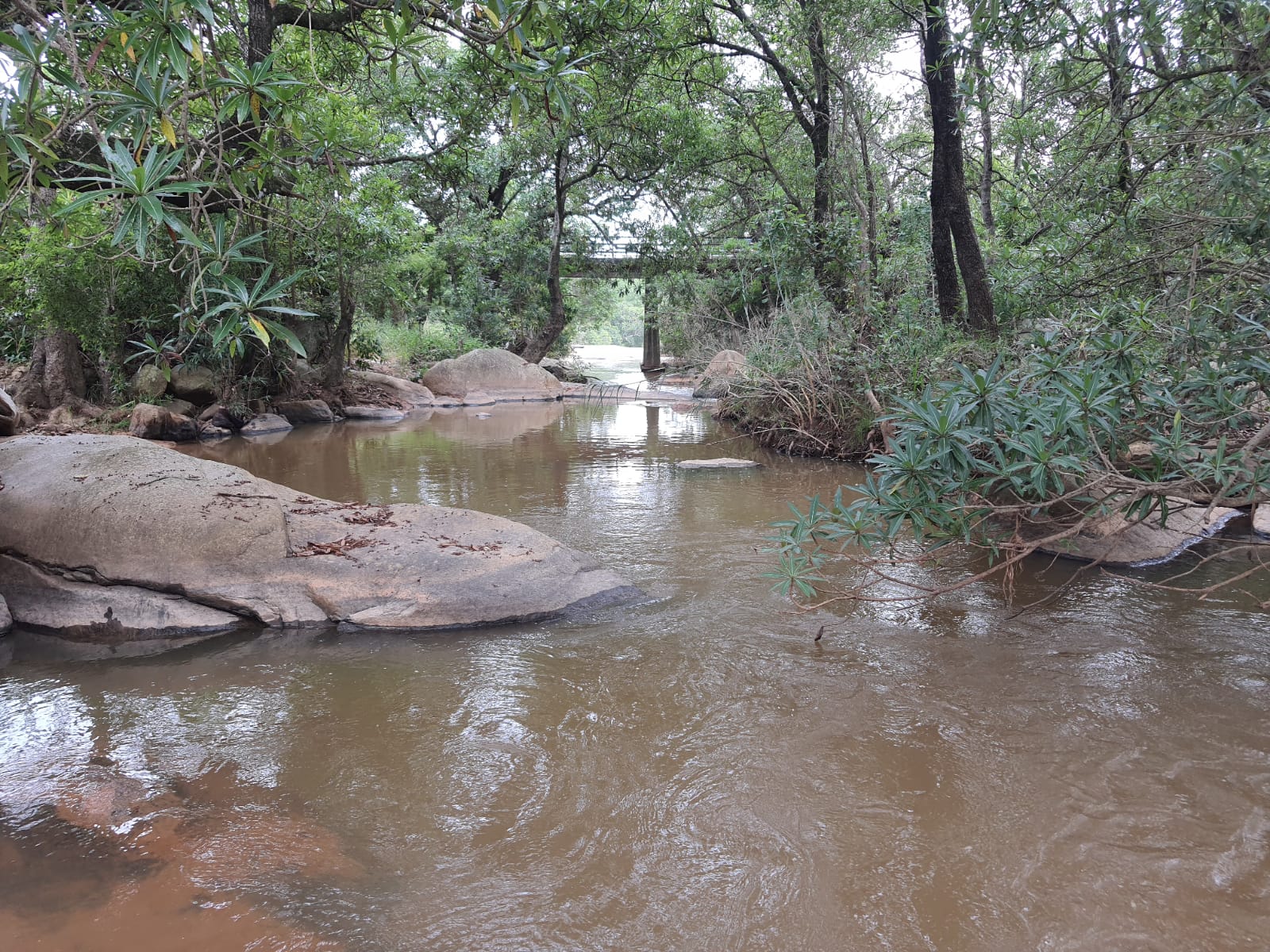 |
| 4 | Tributary 2 (upstream) | This site is impacted by various anthropogenic activities of illegal waste dumping that is received from the surrounding activities. The location of this site next to West Acres residential area exposes this site to the activities that impacts of its habitat and water quality | –25.481455°, 30.962906° | 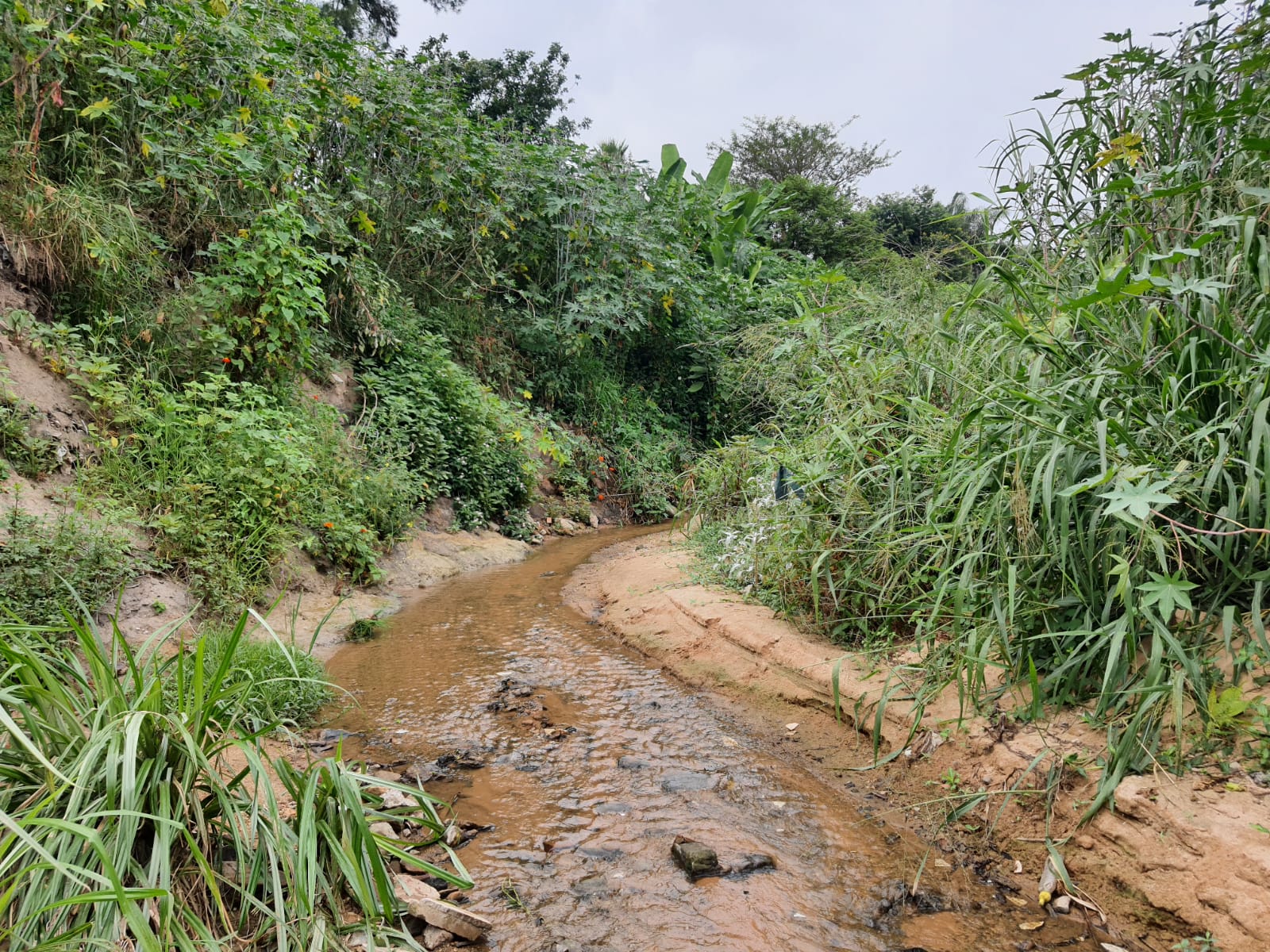 |
| 5 | Tributary 2 (downstream) | This site serves as a receiving site for most of the illegal waste dumping in the area that is taking place next to the braai area and its proximity of being downstream of the town serve a huge disadvantage as water quality and habitat fragmentation are common at this site | –25.463608°, 30.973327° | 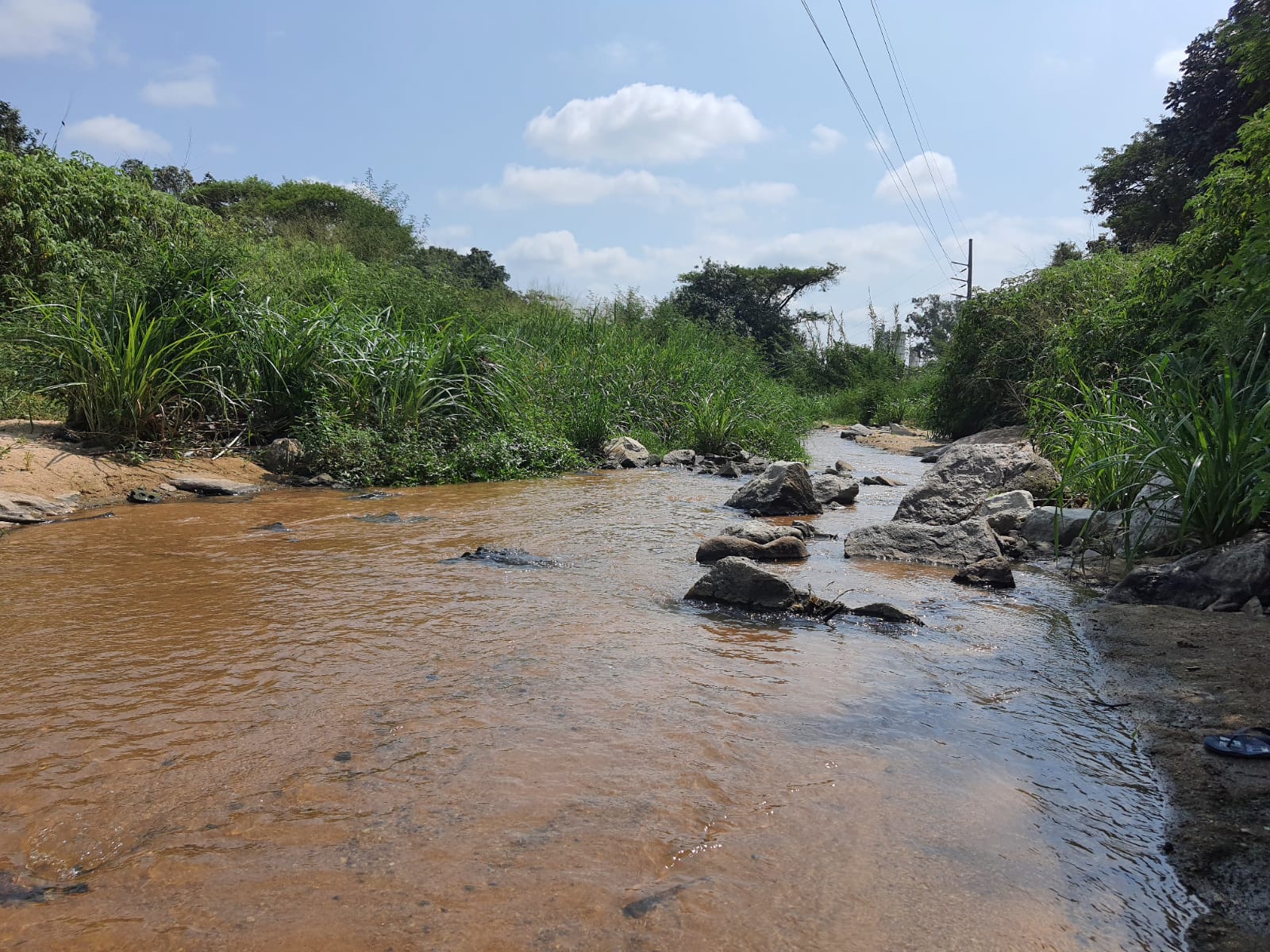 |
| 6 | Tributary 1 (upstream) | at this site, non-treated sewage effluent is flowing into the stream. Illegal domestic waste dumping is also occurring at this site, the location of this site is next to a hospital, and fast–food outlet which contributes to illegal dumping into the stream. | –25.475297°, 30.972200° | 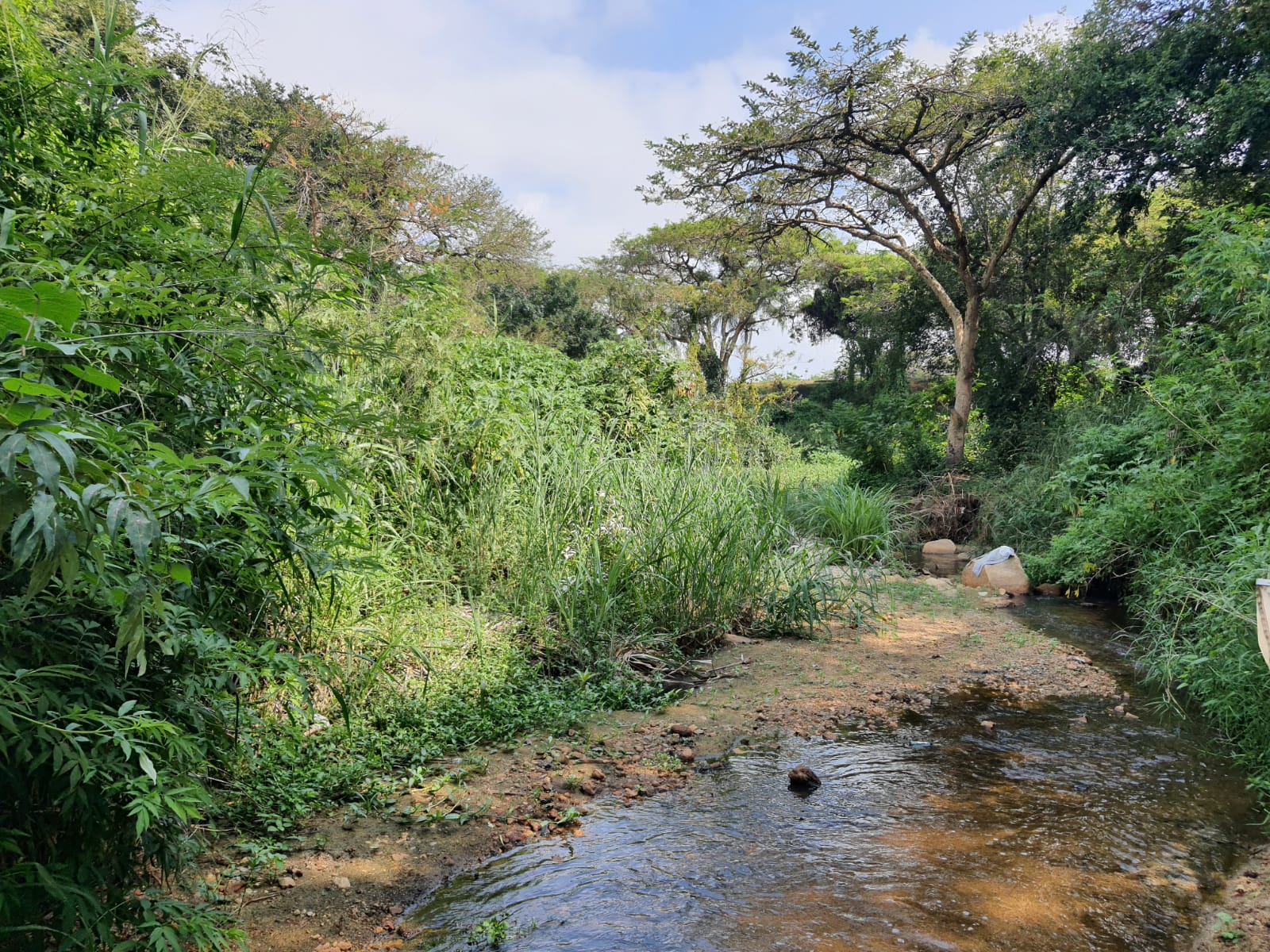 |
| 7 | Nel’s River | At this site, water collection for by construction vehicles is taking place. This site is located downstream of the Elawini Lifestyle Estate | –25.426909°, 30.964544° | 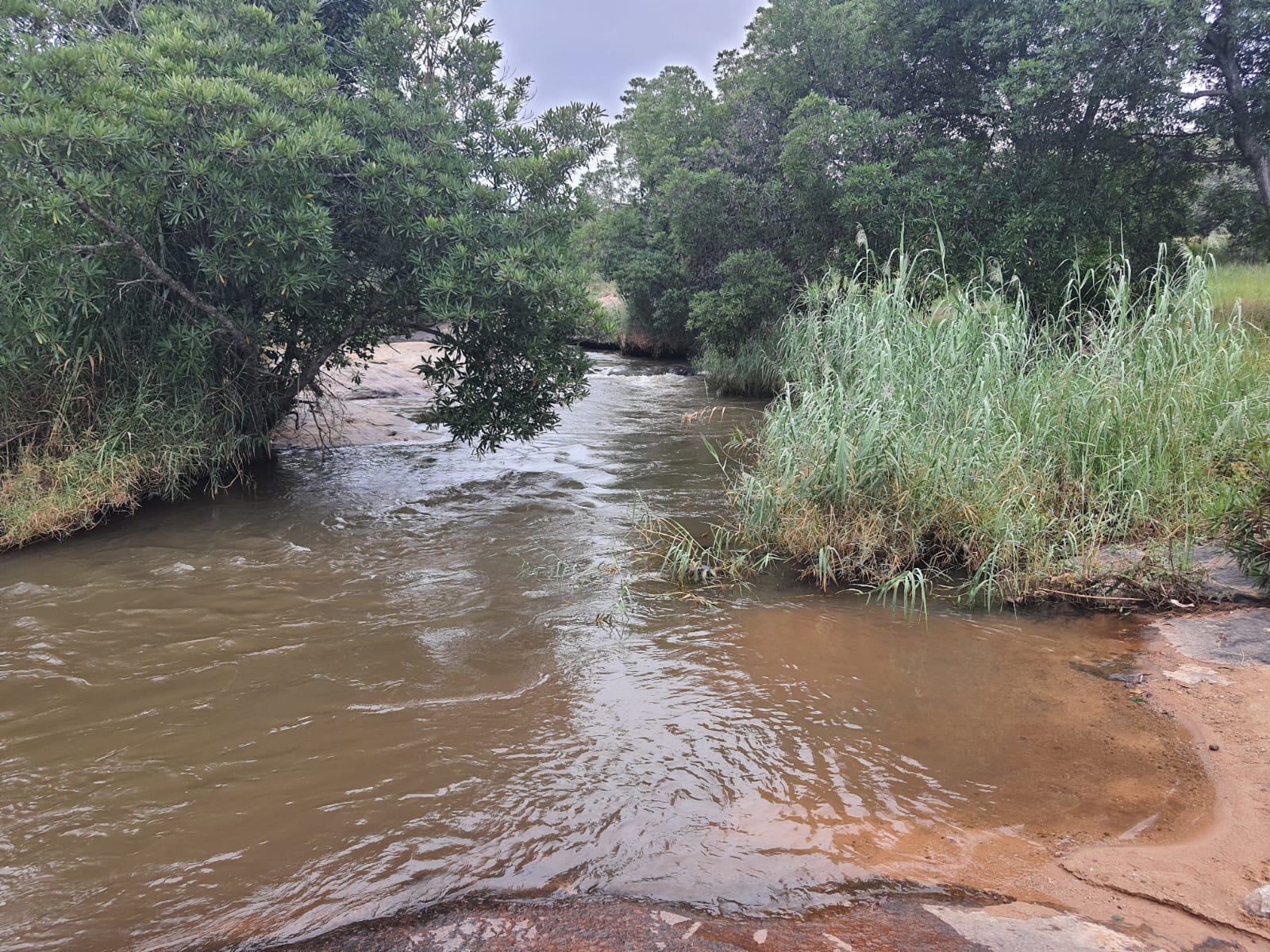 |

**Table S2**. Macroplastic debris type, resin, and abundance per 25 m^2^ found along the Crocodile River across sites during autumn. Abbreviations: Acrylonitrile Butadiene Styrene (ABS), LDPE – low density polyethylene, PET – Polyethylene terephthalate, PS – polystyrene, PVC – polyvinyl chloride, PP – polypropylene, CA – cellulose acetate, HDPE – high–density polyethylene, ABS – acrylonitrile butadiene styrene

| **Plastic fragments** | **Type** | **Rasin Group** | **Site 1** | **Site 2** | **Site 3** | **Site 4** | **Site 5** | **Site 6** | **Site 7** |
| --- | --- | --- | --- | --- | --- | --- | --- | --- | --- |
| Appliance part 1 | Hard | ABS | 6.1 ± 2.2 | 2.7 ± 3.9 |  |  | 12 ± 4.1 | 4.1 ± 5.8 |  |
| Appliance part 2 | Hard | LDPE |  | 2.7 ± 3.9 |  |  | 1.2 ± 1.7 |  |  |
| Bottle cap 1 | Hard | HDPE | 4.5 ± 6.4 | 22.7 ± 16.5 | 18.2 ± 6.8 | 7.2 ± 10.2 | 12.8 ± 7.5 | 20.8 ± 17.6 | 6.1 ± 0.7 |
| Cigarette filter | Hard | CA |  |  |  |  |  |  | 11.1 ± 15.7 |
| Cigarette pack | Film | PP |  | 0.8 ± 1.2 |  |  |  |  |  |
| Deodorant | Hard | HDPE |  | 6.4 ± 6.6 |  |  |  |  |  |
| Detergent bottle 1 | Film | HDPE |  |  |  |  | 2.5 ± 3.5 | 5.5 ± 7.8 |  |
| Detergent bottle 2 | Hard | HDPE | 3.8 ± 5.4 | 0.8 ± 1.2 |  |  |  |  |  |
| Food container 1 | Form | PS |  |  | 3.3 ± 4.7 |  | 2.5 ± 3.5 | 9.7 ± 1.9 | 1.6 ± 2.3 |
| Food container 2 | Foam | PS | 11.3 ± 16 | 5.1 ± 7.3 | 8.5 ± 6.7 | 0.8 ± 1.1 | 4.5 ± 6.4 |  | 6.1 ± 0.7 |
| Food wrapper | Film | PS | 15.3 ± 21.7 | 5.5 ± 7.8 | 8.5 ± 6.7 | 24.1 ± 34.2 | 5 ± 7 |  | 13.3 ± 4.7 |
| Furniture wrapper 1 | Film | LDPE |  | 2.7 ± 3.9 |  |  | 4.5 ± 6.4 |  |  |
| Furniture wrapper 2 | Foam | PS |  | 4.3 ± 6 | 1.9 ± 2.7 |  |  |  |  |
| Gloves | Hard | PVC | 2.2 ± 3.2 | 2.7 ± 3.9 | 1.9 ± 2.7 |  |  |  | 5.5 ± 7.8 |
| Mat | Hard | LDPE |  |  | 1.9 ± 2.7 |  |  |  |  |
| Medicine bottle | Hard | HDPE |  | 0.8 ± 1.2 |  |  |  |  | 3.3 ± 4.7 |
| Milk container | Hard | PP |  |  | 7.1 ± 0.7 | 1.6 ± 2.2 | 1.2 ± 1.7 |  |  |
| Other jug | Hard | PET |  |  |  | 0.8 ± 1.1 | 1.2 ± 1.7 |  |  |
| Pampers | Foam | PS |  |  | 1.9 ± 2.7 |  |  |  |  |
| Perfume bottle | Hard | HDPE |  |  |  |  |  |  | 1.6 ± 2.3 |
| Pipe | Hard | PVC |  |  |  | 12.5 ± 17.6 |  |  | 3.3 ± 4.7 |
| Plastic bag | Film | LDPE | 22.2 ± 12.1 | 8.3 ± 11.7 | 24.8 ± 2.5 | 25.8 ± 34.2 | 10.7 ± 2.4 | 34.7 ± 33.3 | 14.4 ± 12.5 |
| Plastic bottle | Hard | PET | 19.7 ± 17 | 30 ± 11.1 | 9.6 ± 13.5 | 18.1 ± 9.6 | 18.6 ± 12.2 | 20.8 ± 17.6 | 16.1 ± 14.9 |
| Plastic cup 1 | Film | LDPE |  |  |  | 4.8 ± 6.8 | 3.7 ± 5.3 |  |  |
| Plastic cup 2 | Foam | PS |  |  | 10 ± 14.1 |  |  |  |  |
| Plastic cutlery | Hard | PP |  |  |  | 2.4 ± 3.4 |  |  | 8.3 ± 11.7 |
| Plastic lid | Hard | PP |  |  |  | 1.6 ± 2.2 | 1.2 ± 1.7 |  | 1.6 ± 2.3 |
| Plastic rope | Film | PVC | 2.2 ± 3.2 | 0 ± 0 | 1.9 ± 2.7 |  |  |  | 1.6 ± 2.3 |
| Sack | Film | PP | 7.6 ± 10.8 |  |  |  | 4.5 ± 6.4 |  | 5.5 ± 7.8 |
| Shaver | Hard | HDPE |  |  |  |  | 1.2 ± 1.7 |  |  |
| Shoe | Hard | LDPE | 2.2 ± 3.2 | 2.7 ± 3.9 |  |  | 3.7 ± 5.3 |  |  |
| Soap wrapper | Film | LDPE |  |  |  |  | 5.7 ± 4.6 |  |  |
| Strapping tape | Hard | PP | 2.2 ± 3.2 |  |  |  | 1.2 ± 1.7 | 4.1 ± 5.8 |  |
| Toothpaste tube | Hard | LDPE |  |  |  |  | 1.2 ± 1.7 |  |  |
| Yogurt container | Hard | PS |  | 0.8 ± 1.2 |  |  |  |  |  |

**Table S3**. Macroplastic debris type, resin, and abundance per 25 m^2^ found along the Crocodile River across sites during winter. Abbreviations: LDPE – low density polyethylene, PET – Polyethylene terephthalate, PS – polystyrene, PVC – polyvinyl chloride, PP – polypropylene, CA – cellulose acetate, HDPE – high–density polyethylene, ABS – acrylonitrile butadiene styrene

| **Plastic fragments** | **Type** | **Rasin Group** | **Site 1** | **Site 2** | **Site 3** | **Site 4** | **Site 5** | **Site 6** | **Site 7** |
| --- | --- | --- | --- | --- | --- | --- | --- | --- | --- |
| Appliance part 1 | Hard | ABS |  |  | 2.6 ± 3.7 | 4 ± 4.3 | 15.3 ± 21.7 |  | 5 ± 7 |
| Appliance part 2 | Hard | LDPE |  |  | 4.3 ± 1.2 | 0.4 ± 0.7 |  |  |  |
| Bottle cap 1 | Hard | HDPE |  |  | 22.5 ± 16.9 | 2.4 ± 3.5 | 11.5 ± 16.3 | 6.2 ± 8.8 | 12.5 ± 10.6 |
| Bottle cap 2 | Hard | HDPE |  |  | 3.4 ± 4.8 |  |  |  |  |
| Bubble blower | Hard | PP |  |  | 1.7 ± 2.4 |  |  |  |  |
| Cigarette filter | Hard | CA |  |  | 10.3 ± 14.6 |  |  |  | 5 ± 7 |
| Cigarette pack | Film | PP |  |  |  | 0.9 ± 1.4 | 3.8 ± 5.4 |  | 5 ± 7 |
| Deodorant | Hard | HDPE |  | 15.6 ± 9.2 |  |  |  |  |  |
| Food container 1 | Form | PS |  | 10.1 ± 1.4 |  |  | 25 ± 35.3 |  | 5 ± 7 |
| Food container 2 | Foam | PS |  | 36.3 ± 51.4 | 1.7 ± 2.4 | 7.6 ± 9.4 |  | 22.3 ± 13.9 | 20 ± 0 |
| Food wrapper | Film | PS | 35.7 ± 50.5 | 10.1 ± 1.4 | 14.8 ± 16.1 | 48.5 ± 18.1 | 7.6 ± 10.8 | 6.2 ± 8.8 | 12.5 ± 17.6 |
| Furniture wrapper 1 | Film | LDPE |  |  |  | 4.9 ± 7 | 12.5 ± 17.6 |  |  |
| Furniture wrapper 2 | Foam | PS |  |  |  |  | 12.5 ± 17.6 | 6.4 ± 9.1 |  |
| Juice box | Hard | PP |  |  |  | 0.4 ± 0.7 |  |  |  |
| Motor oil bottle | Hard | HDPE |  |  | 1.7 ± 2.4 | 4 ± 4.3 |  |  |  |
| Peanut butter container | Hard | PET |  |  |  | 0.4 ± 0.7 |  |  |  |
| Perfume bottle | Hard | HDPE |  |  |  |  | 3.8 ± 5.4 |  |  |
| Plastic bag | Film | LDPE | 40 ± 56.5 | 5.5 ± 7.8 | 16.6 ± 13.7 | 16.7 ± 16.7 |  | 20.3 ± 24.2 | 15 ± 21.2 |
| Plastic bottle | Hard | PET | 14.2 ± 20.2 | 5.5 ± 7.8 | 7.8 ± 11.1 | 2.9 ± 4.2 | 3.8 ± 5.4 | 30.4 ± 25.3 | 12.5 ± 10.6 |
| Plastic cup 1 | Film | LDPE |  |  | 3.4 ± 4.8 |  |  |  |  |
| Plastic cup 2 | Foam | PS |  | 5.5 ± 7.8 |  | 0.4 ± 0.7 |  | 1.6 ± 2.2 |  |
| Plastic lid | Hard | PP |  |  | 3.4 ± 4.8 |  |  |  |  |
| Plastic rope | Film | PVC |  |  | 2.6 ± 3.7 |  |  |  |  |
| Plastic straw | Film | PP |  |  |  | 1.4 ± 2.1 |  |  |  |
| Red tape | Hard | PVC |  |  |  | 0.4 ± 0.7 |  |  |  |
| Soap wrapper | Film | LDPE | 10 ± 14.1 | 5.5 ± 7.8 |  |  | 3.8 ± 5.4 |  |  |
| Strapping tape | Hard | PP |  |  |  |  |  | 6.2 ± 8.8 | 2.5 ± 3.5 |
| Umbrella | Film | PET |  |  | 2.6 ± 3.7 |  |  |  |  |
| Vaseline container | Hard | PP |  | 5.5 ± 7.8 |  |  |  |  |  |
| Yogurt container | Hard | PS |  |  |  | 3.5 ± 5 |  |  |  |

**Table S4.** Macroplastic debris type, resin, and abundance per 25 m^2^ found along the Crocodile River across sites during spring. Abbreviations: LDPE – low density polyethylene, PET – Polyethylene terephthalate, PS – polystyrene, PVC – polyvinyl chloride, PP – polypropylene, CA – cellulose acetate, HDPE – high–density polyethylene, ABS – acrylonitrile butadiene styrene

| **Plastic fragments** | **Type** | **Rasin Group** | **Site 1** | **Site 2** | **Site 3** | **Site 4** | **Site 5** | **Site 6** | **Site 7** |
| --- | --- | --- | --- | --- | --- | --- | --- | --- | --- |
| Appliance part 1 | Hard | ABS |  |  |  |  |  |  | 12.8 ± 13.1 |
| Appliance part 2 | Hard | LDPE |  |  |  |  |  |  | 1.7 ± 2.5 |
| Bottle cap 1 | Hard | HDPE | 4.1 ± 5.8 | 11.5 ± 16.3 | 14.5 ± 6.3 | 7.5 ± 2.2 |  | 29.4 ± 13.3 | 20.2 ± 18.5 |
| Cider plastic | Film | PS |  |  | 5 ± 7 | 2.2 ± 3.1 |  |  | 5.3 ± 7.5 |
| Cigarette filter | Hard | CA |  |  | 2.5 ± 3.5 |  | 18.7 ± 26.5 |  |  |
| Cigarette pack | Film | PP | 10 ± 14.1 |  | 2.5 ± 3.5 | 2.2 ± 3.1 |  |  |  |
| Detergent bottle 2 | Hard | HDPE |  |  |  |  |  | 3.3 ± 4.7 |  |
| Ear bud tips | Hard | PU |  |  |  | 2.2 ± 3.1 |  |  |  |
| Elastic Band | Hard | LDPE | 10 ± 14.1 |  |  |  |  |  |  |
| Floss | Hard | HDPE | 10 ± 14.1 |  |  |  |  |  |  |
| Food container 1 | Form | PS | 0 ± 0 |  | 2.3 ± 3.3 |  |  | 3.3 ± 4.7 |  |
| Food container 2 | Foam | PS | 0 ± 0 | 10 ± 14.1 | 17 ± 2.8 | 2.2 ± 3.1 |  | 6.1 ± 0.7 | 5.3 ± 7.5 |
| Food wrapper | Film | PS | 28.3 ± 16.4 |  | 24.5 ± 7.7 | 35.2 ± 11.3 |  | 3.3 ± 4.7 | 23.4 ± 17.3 |
| Furniture wrapper 1 | Film | LDPE | 4.1 ± 5.8 |  | 2.3 ± 3.3 | 24.2 ± 30 |  | 6.6 ± 9.4 |  |
| Globe holder | Hard | PVC |  |  | 2.3 ± 3.3 |  |  |  |  |
| Glue container | Hard | PET |  |  |  | 0.7 ± 1 |  |  |  |
| Lunchbox lid | Hard | PP |  |  |  |  | 3.1 ± 4.4 |  |  |
| Mask | Foam | PP |  |  | 2.3 ± 3.3 |  |  |  |  |
| Medicine bottle | Hard | HDPE |  | 7.6 ± 10.8 |  |  |  |  |  |
| Packaging plastic | Film | LDPE |  |  |  |  |  | 3.3 ± 4.7 |  |
| Plastic bag | Film | LDPE | 12.5 ± 17.6 | 17.6 ± 3.2 | 14.5 ± 6.3 | 2.2 ± 3.1 | 6.2 ± 8.8 | 2.7 ± 3.9 | 5.3 ± 7.5 |
| Plastic bottle | Hard | PET | 8.3 ± 11.7 | 33 ± 18.4 | 4.8 ± 0.1 | 13.5 ± 6.5 | 22.9 ± 14.7 | 32.2 ± 17.2 | 22 ± 15.9 |
| Plastic cup 1 | Film | LDPE |  |  |  |  |  |  |  |
| Plastic cup 2 | Foam | PS |  |  | 2.5 ± 3.5 |  |  |  |  |
| Plastic cutlery | Hard | PP |  |  | 2.5 ± 3.5 | 2.2 ± 3.1 | 6.2 ± 8.8 |  |  |
| Plastic hanger | Hard | ABS |  | 10 ± 14.1 |  |  |  |  |  |
| Plastic rope | Film | PVC | 4.1 ± 5.8 |  |  |  |  | 3.3 ± 4.7 |  |
| Plastic straw | Film | PP |  |  |  | 2.2 ± 3.1 |  |  |  |
| Pregnancy test stick | Hard | HDPE |  |  |  | 2.2 ± 3.1 |  |  |  |
| Sack | Film | PP |  |  |  |  | 16.6 ± 23.5 | 6.1 ± 0.7 |  |
| Sanitizer bottle | Hard | PP |  |  |  |  | 6.2 ± 8.8 |  |  |
| Shoe | Hard | LDPE | 4.1 ± 5.8 |  |  |  | 3.1 ± 4.4 |  |  |
| Shoe sole | Hard | PVC |  | 10 ± 14.1 |  |  |  |  |  |
| Strapping tape | Hard | PP | 4.1 ± 5.8 |  |  |  |  |  |  |
| Vaseline container | Hard | PP |  |  |  |  |  |  | 1.7 ± 2.5 |
| Yogurt container | Hard | PS |  |  |  |  | 16.6 ± 23.5 |  | 1.7 ± 2.5 |

**Table S5.** Macroplastic debris type, resin, and abundance per 25 m^2^ found along the Crocodile River across sites during summer. Abbreviations: LDPE – low density polyethylene, PET – Polyethylene terephthalate, PS – polystyrene, PVC – polyvinyl chloride, PP – polypropylene, CA – cellulose acetate, HDPE – high–density polyethylene, ABS – acrylonitrile butadiene styrene

| **Plastic fragments** | **Type** | **Rasin Group** | **Site 1** | **Site 2** | **Site 3** | **Site 4** | **Site 5** | **Site 6** | **Site 7** |
| --- | --- | --- | --- | --- | --- | --- | --- | --- | --- |
| Appliance part 1 | Hard | ABS |  |  |  |  |  |  | 7.6 ± 10.8 |
| Appliance part 2 | Hard | LDPE |  |  |  |  |  |  | 3.8 ± 5.4 |
| Bottle cap 1 | Hard | HDPE | 19.6 ± 19.4 | | 2.5 ± 3.5 | 5.8 ± 8.3 | 3.8 ± 5.4 |  | 4.8 ± 3.9 |
| Bottle cap 2 | Hard | HDPE |  |  | 15 ± 21.2 |  |  |  |  |
| Cider plastic | Film | PS |  |  |  | 0.9 ± 1.3 | 0 ± 0 | 1.6 ± 2.2 | 0 ± 0 |
| Cigarette pack | Film | PP |  |  | 5 ± 7 |  |  |  |  |
| Deodorant | Hard | HDPE |  |  | 2.5 ± 3.5 |  |  |  |  |
| Food container 1 | Form | PS | 2.9 ± 4.1 | 21.2 ± 17.1 | 6.6 ± 9.4 | 0.9 ± 1.3 |  |  | 1 ± 1.4 |
| Food container 2 | Foam | PS | 2.9 ± 4.1 |  | 2.5 ± 3.5 | 5.8 ± 8.3 |  | 10.7 ± 15.1 | |
| Food wrapper | Film | PS | 17.6 ± 24.9 | 34.8 ± 2.1 | 15.8 ± 15.3 | 37.9 ± 10.1 | 7.6 ± 10.8 | 45.9 ± 5.7 | 30.6 ± 43.2 |
| Furniture wrapper 1 | Film | LDPE | 16.6 ± 23.5 | 9 ± 12.8 |  |  | 8.8 ± 1.6 | 11.2 ± 15.9 | 1 ± 1.4 |
| Gloves | Hard | PVC |  |  |  |  | 3.8 ± 5.4 |  |  |
| Glue container | Hard | PET | 16.6 ± 23.5 | |  |  |  |  |  |
| Milk container | Hard | PP |  |  | 12.5 ± 17.6 | |  |  | 4.8 ± 3.9 |
| Motor oil bottle | Hard | HDPE |  |  |  | 3.8 ± 5.4 |  |  |  |
| Packaging plastic | Film | LDPE | 2.9 ± 4.1 |  | 3.3 ± 4.7 |  |  |  | 3.8 ± 5.4 |
| Perfume bottle | Hard | HDPE |  | 16.6 ± 23.5 | | 8.8 ± 12.4 |  |  |  |
| Plastic bag | Film | LDPE | 8.8 ± 12.4 | 13.6 ± 19.2 | 8.3 ± 2.3 | 22.1 ± 23 | 46.5 ± 33.1 | 20.7 ± 11 | 33.2 ± 18.2 |
| Plastic bottle | Hard | PET | 11.7 ± 16.6 | 4.5 ± 6.4 | 25.8 ± 29.4 | 11.5 ± 16.3 | 16.5 ± 9.2 | 9.6 ± 13.6 | 7.9 ± 0.3 |
| Plastic cup 2 | Foam | PS |  |  |  |  |  |  | 1 ± 1.4 |
| Pregnancy test stick | Hard | HDPE |  |  |  | 0.9 ± 1.3 |  |  |  |
| Sack | Film | PP |  |  |  |  | 8.8 ± 1.6 |  |  |
| Shoe sole | Hard | PVC |  |  |  |  | 3.8 ± 5.4 |  |  |
| Yogurt container | Hard | PS |  |  |  | 0.9 ± 1.3 |  |  |  |
